# *N*,*N*-Dimethylation of nitrobenzenes with CO_2_ and water by electrocatalysis†
†Electronic supplementary information (ESI) available. See DOI: 10.1039/c7sc01058c



**DOI:** 10.1039/c7sc01058c

**Published:** 2017-06-07

**Authors:** Xiaofu Sun, Qinggong Zhu, Jiayin Hu, Xinchen Kang, Jun Ma, Huizhen Liu, Buxing Han

**Affiliations:** a Beijing National Laboratory for Molecular Sciences , Key Laboratory of Colloid and Interface and Thermodynamics , Institute of Chemistry , Chinese Academy of Sciences , Beijing 100190 , P. R. China . Email: hanbx@iccas.ac.cn; b University of Chinese Academy of Sciences , Beijing 100049 , P. R. China

## Abstract

We have proposed a strategy for the synthesis of *N*,*N*-dimethylanilines from nitrobenzene and its derivatives, CO_2_, and water *via* an electrochemical reaction under ambient conditions. H^+^ generated from H_2_O was used as the hydrogen source. Pd/Co–N/carbon, in which the Pd nanoparticles were supported on Co–N/carbon, was designed and used as the electrocatalyst. It was found that the electrocatalyst was very efficient for the reaction in MeCN solution with 1-butyl-3-methylimidazolium bis(trifluoromethylsulfonyl)imide ([Bmim]Tf_2_N) as the supporting electrolyte and 1-amino-methylphosphonic acid (AMPA) as the thermal co-catalyst. A series of control experiments showed that Pd/Co–N/carbon and AMPA cooperated very well in accelerating the reaction. This synthetic route has some obvious advantages, such as using CO_2_ and water as the reactants, ambient reaction conditions, and high yields of the desired products. This opens up a way to synthesize chemicals by the combination of an electrocatalyst and a thermal catalyst with organic compounds, CO_2_, and water as the reactants.

## Abstract 2

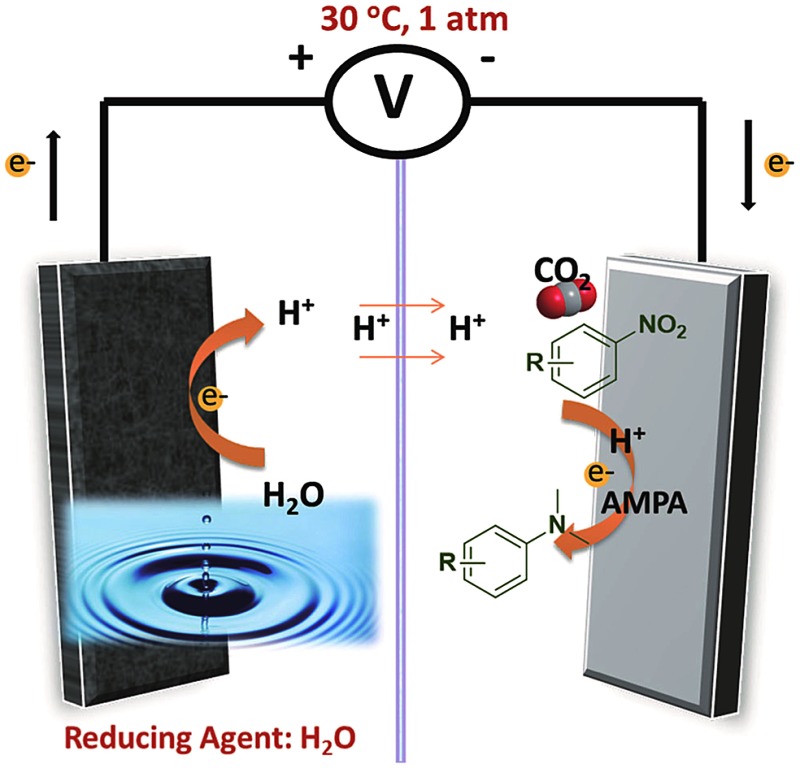
We have developed a strategy for the synthesis of *N*,*N*-dimethylanilines by the *N*,*N*-dimethylation of nitrobenzenes with CO_2_ using H_2_O as the hydrogen source.

## Introduction

Carbon dioxide (CO_2_) is an abundant, inexpensive, harmless and renewable C1 resource.^1,2^ CO_2_ chemistry has emerged as one of the most significant branches of chemistry. Owing to its thermodynamic stability and kinetic inertness, a well designed activation of CO_2_ and a thermodynamic driving force are required for efficient conversion.^3–5^ The transformation of CO_2_ into different chemicals has been reported, such as the direct hydrogenation of CO_2_ to organic acids and alcohols.^6,7^ In parallel, the direct introduction of CO_2_ into organic substrates is also an attractive goal in modern chemistry,^8–11^ and the exploration of new routes is very interesting.

*N*-Methylation and *N*,*N*-dimethylation reactions are very important in the chemical industry, as they can obtain a series of useful intermediates for the synthesis of many valuable products, such as dyes, pesticides and perfumes.^12–20^ Traditionally, they can be synthesized through the methylation of amines with methanol or formaldehyde.^19,20^ Using CO_2_ as the C1 resource is a very promising route for the synthesis of *N*,*N*-dimethylanilines. In general, H_2_ or PhSiH_3_ has been used as the reducing agent for the *N*-methylation reaction of anilines.^12–18^ Anilines are usually synthesized by the hydrogenation of nitrobenzenes. Recently, the direct *N*-methylation of nitrobenzenes has also been studied using H_2_ as the reducing agent at high temperature and under high pressure (Fig. S1†).^17^ Obviously, searching for new reducing agents and performing the methylation reaction of nitrobenzenes under mild conditions are desirable.

The electrochemical method has been used to synthesize organic molecules in the past century.^21–25^ It possesses some obvious advantages, such as mild conditions, high functional group tolerance, and innate scalability and sustainability.^21^ Water is an ideal hydrogen source. H^+^ can be obtained *via* the oxygen evolution reaction (OER) at the anode electrode and transferred to the cathode electrode.^26^ It is well known that the efficiency and selectivity of electrochemical reactions depend strongly on the properties of the electrodes and electrolytes, and their coupling.^22^ Therefore, designing a suitable electrocatalyst with abundant active sites and high electrical conductivity is the key to promoting electrocatalytic reactions.

The use of CO_2_ and water as the reactants simultaneously in organic reactions is very attractive. Herein, we have developed a strategy for the synthesis of *N*,*N*-dimethylanilines by the *N*,*N*-dimethylation of nitrobenzene or its derivatives with CO_2_ using H_2_O as the hydrogen source. In the reactions, an electrochemical reaction and thermal reaction were combined with Pd/Co–N/carbon as the electrocatalyst and 1-amino-methylphosphonic acid (AMPA) as the thermal catalyst. It was demonstrated that Pd/Co–N/carbon and AMPA had excellent synergistic effects and the reactions proceeded efficiently under ambient conditions and high yields of the desired products could be reached. As far as we know, this is the first work on the synthesis of *N*,*N*-dimethylanilines using nitrobenzene (or its derivatives), CO_2_, and water as the reactants.

## Results and discussion

The H-type reaction cell used was composed of a cathode, a platinum anode, and an Ag/Ag^+^ reference electrode (Fig. S2†), which was similar to that utilized in the previous works for the electroreduction of CO_2_.^27–31^ The cathode and anode compartments were separated by a Nafion 117 proton exchange membrane (PEM). H_2_SO_4_ aqueous solution was used as the anodic electrolyte. In the reaction, H^+^ from water could be transferred from the anode compartment to the cathode compartment through the PEM, which acted as the hydrogen source for the reaction. The detailed description of the apparatus is given in the ESI.†

Pd is an efficient electrocatalyst for the reduction of CO_2_ to CO or formate.^32,33^ It is known that nanoporous carbon materials have high specific surface areas, high chemical and thermal stabilities, and good conductivities.^34,35^ Zeolitic imidazolate frameworks (ZIFs) have been reported to be outstanding carbon precursors.^36,37^ They can incorporate N atoms and metal species into the carbon lattice, which can enhance the electric conductivity and electron-donor tendency, and act as the hard template during the carbonization process.^36^ Meanwhile, the residual metals can also act as the co-catalyst. We have designed a new electrocatalyst, Pd/Co–N/carbon, using a ZIF as the carbon and nitrogen precursor, and the detailed preparation procedures are given in the ESI.† Very briefly, the Co–N/carbon support was first prepared by the carbonization of the Co–ZIF/graphene oxide (GO) at 800 °C under an Ar atmosphere. The Pd_*x*_/Co–N/carbon electrocatalysts were obtained by immobilizing Pd nanoparticles on the support, in which *x* denotes the average size of the Pd particles in nm. The size of the Pd nanoparticles could be controlled by the reduction temperature and the ratio of sodium citrate (stabilizing agent) to PdCl_2_. The size distributions of the Pd nanoparticles in different catalysts are shown in Fig. S3,† and were obtained from counting more than 200 particles in the transmission electron microscopy (TEM) images. The support had a specific surface area of 380 m^2^ g^–1^, which was determined by the N_2_ adsorption/desorption method. The elemental analysis of the Co–N/carbon support was conducted by inductively coupled plasma optical emission spectroscopy (ICP-OES). The results showed that the content of Co, C and N elements in the support is 37.69 wt%, 30.73 wt% and 1.05 wt%, respectively. The surface composition of the support detected by X-ray photoelectron spectroscopy (XPS) is given in Table S1.†

**Fig. 1:**
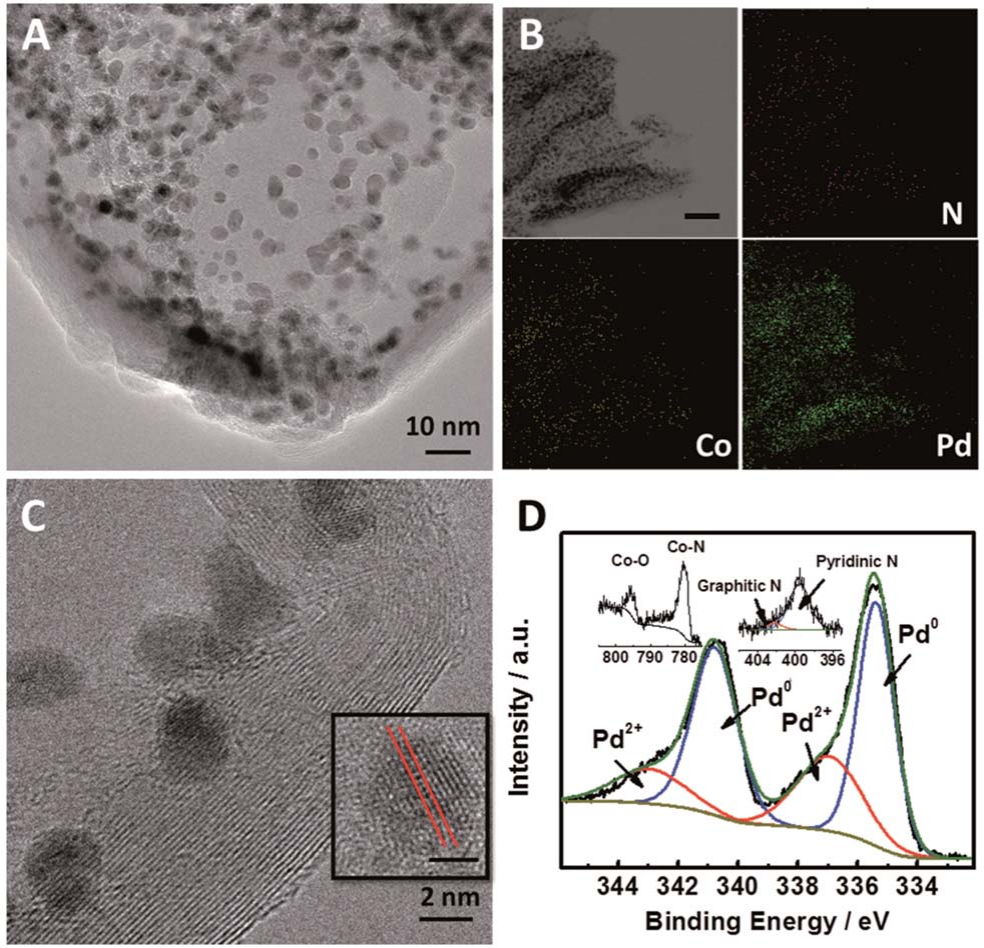
Structural and elemental analysis of Pd_2.2_/Co–N/carbon. (A) TEM image of Pd_2.2_/Co–N/carbon. (B) Corresponding elemental mappings of Pd_2.2_/Co–N/carbon (scale bar: 50 nm). (C) HR-TEM image of Pd_2.2_/Co–N/carbon. The scale bar in the inset of (C) is 1 nm. (D) XPS spectra of Pd 3d, Co 2p and N 1s orbitals of Pd_2.2_/Co–N/carbon.

Fig. 1 shows high-resolution transmission electron microscopy (HR-TEM) images of Pd_2.2_/Co–N/carbon. It can be seen that the Pd nanoparticles were uniformly deposited on the Co–N/carbon support (Fig. 1A). Elemental distribution mappings also illustrate the co-existence of Pd and Co elements and the Pd nanoparticles were dispersed on the support homogeneously (Fig. 1B). The typical Pd (111) plane with the characteristic lattice spacing of 0.23 nm could be observed (Fig. 1C). XPS spectra (Fig. 1D) show the chemical nature of the catalyst, including the peaks belonging to Pd^0^ (Pd 3d: 340.7 and 335.4 eV), Pd^2+^ (Pd 3d: 343.0 and 336.9 eV), Co–N (Co 2p: 780.1 eV), Co–O (Co 2p: 795.4 eV), pyridinic N (N 1s: 399.5 eV) and graphitic N (N 1s: 402.2 eV).^36,38^ The results provided direct evidence that most of the Pd nanoparticles existed in the form of Pd^0^, and some electrochemical active sites for CO_2_ reduction such as the pyridinic N and Co–N_*x*_ moieties^39,40^ existed in the support. Furthermore, the actual Pd loadings in all of the catalysts were 18.3 ± 0.6 wt%, as measured by ICP-OES as listed in Table S2.† TEM and HR-TEM images of the other Pd_*x*_/Co–N/carbon catalysts with different Pd sizes are shown in Fig. S4.†

To prepare the electrodes, the Pd_*x*_/Co–N/carbon catalysts were suspended in acetone with Nafion D-521 dispersion to form a homogeneous ink with the aid of ultrasound, which was spread onto carbon paper (CP) to obtain the working electrodes. Ionic liquids (ILs) are efficient supporting electrolytes for the reduction of CO_2_.^27,41^ The initial screening was performed for the methylation reaction of nitrobenzene, **1a**, with CO_2_ and H^+^ from H_2_O over Pd_2.2_/Co–N/carbon, using MeCN containing 0.5 M 1-butyl-3-methylimidazolium bis(trifluoromethylsulfonyl)imide ([Bmim]Tf_2_N) as the electrolyte, in the presence of AMPA. As expected, the reduction potential was crucial for the conversion of **1a** (Table 1, entries 1–6). The most effective potential was –2.3 V *vs.* Ag/Ag^+^, and afforded the full conversion of **1a** with a 92% yield of *N*,*N*-dimethylaniline **1d** at 30 °C. Lowering the reaction temperature to 20 °C resulted in a yield of only 65% after 10 h (Table S3†). Increasing the temperature to 40 °C, 50 °C and 60 °C resulted in 81%, 72% and 58% yields of **1d** (Table S3†) due mainly to the lower solubility of CO_2_ in the electrolyte. Therefore, 30 °C is an optimal temperature. Table 1 (entries 5 and 7–10) and Fig. S5† indicated that reactant **1a** could be converted completely after 10 h. Subsequently, the conversion of **1a** was also studied using MeCN containing other ILs as the supporting electrolytes, but their performances were not as good as that of [Bmim]Tf_2_N (Table 1, entries 5 and 11–16). Furthermore, the solutions of [Bmim]Tf_2_N with AMPA in DMSO, DMF, MeNO_2_ and 1,4-dioxane were also used as the electrolytes, and lower yields of **1d** were obtained (Table S4†). The results indicated that [Bmim]Tf_2_N in MeCN solution was the best electrolyte.

**Table 1:** Electrocatalytic methylation of nitrobenzene with CO_2_ and water over Pd_2.2_/Co–N/carbon^*a*^

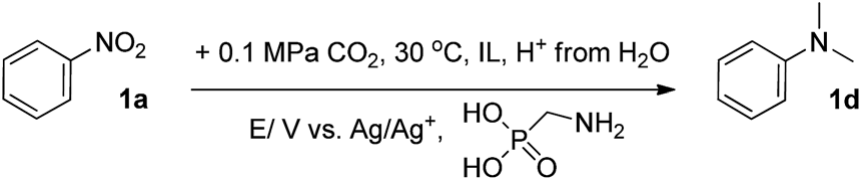				
Entry	Electrolyte^*b*^	*E* ^*c*^/V	*t*/h	Yield^*d*^/%
1	[Bmim]Tf_2_N	–1.9	10	13
2	[Bmim]Tf_2_N	–2.0	10	36
3	[Bmim]Tf_2_N	–2.1	10	68
4	[Bmim]Tf_2_N	–2.2	10	89
5	[Bmim]Tf_2_N	–2.3	10	92
6	[Bmim]Tf_2_N	–2.4	10	92
7	[Bmim]Tf_2_N	–2.3	1	7
8	[Bmim]Tf_2_N	–2.3	5	56
9	[Bmim]Tf_2_N	–2.3	8	85
10	[Bmim]Tf_2_N	–2.3	12	92
11	[Bmim]PF_6_	–2.3	10	81
12	[Bmim]BF_4_	–2.3	10	82
13	[Bmim]TfO	–2.3	10	85
14	[Bmim]ClO_4_	–2.3	10	23
15	[Bmim]NO_3_	–2.3	10	15
16	[Bmim]H_2_PO_4_	–2.3	10	30

^*a*^Reaction conditions: nitrobenzene (1.0 mmol), AMPA (0.06 mmol), CO_2_ (0.1 MPa) and 30 °C.

^*b*^Electrolyte (30 mL) is CO_2_-saturated MeCN, containing 0.5 M IL.

^*c*^All potentials are reported with respect to Ag/Ag^+^.

^*d*^Yields determined by ^1^H NMR spectroscopy.

Fig. S6† summarizes the yields of **1d** from the electrochemical conversion of **1a** over the Pd_*x*_/Co–N/carbon catalysts with different Pd particle sizes at different potentials. The electronic properties of the nanoparticles can be tuned by controlling the sizes.^42,43^ Generally, smaller nanoparticles have lower d-band centers, which in turn result in a decrease in their adsorption energies.^44^ With respect to the reduction of CO_2_, lowering the d-band center reduces the binding energy of the hypothetical intermediates,^45^ thereby enhancing the rate of CO_2_ reduction and the conversion of nitrobenzene.

The methylation of various substituted nitrobenzenes over Pd_2.2_/Co–N/carbon was investigated (Table 2). The electron-donating methyl, methoxy and methylthio groups in the 4-position of the aromatic ring of **1a** reduced the reactivity giving yields of 75% (**2d**), 74% (**3d**) and 71% (**4d**), respectively (Table 2, entries 1–3). For halogen-substituted nitrobenzenes, the desired products were obtained in 82–90% yields (Table 2, entries 4–8). When 4-nitrobiphenyl **10a** and 2-nitrobiphenyl **11a** were used, the corresponding products were generated in 78% (**10d**) and 76% (**11d**) yields, respectively (Table 2, entries 9 and 10). Di-substituted nitrobenzenes such as 5-nitro-*m*-xylene (**12a**), 2,6-dimethylnitrobenzene (**13a**) and 3-methyl-4-nitroanisole (**14a**) also showed high reactivity, which gave the desired products in 75–82% yields (Table 2, entries 11–13). To our delight, the *N*,*N*-dimethylation of benzonitrile and its derivatives could also be carried out with moderate yields (Table S5†).

**Table 2:** Electrocatalytic methylation of substituted nitrobenzenes with CO_2_ and water over Pd_2.2_/Co–N/carbon^*a*^

Entry	Substrates	Products	Yield^*b*^/%
1	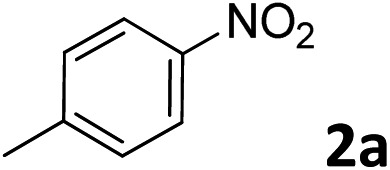	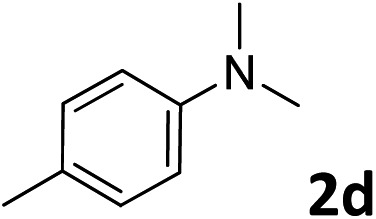	75
2	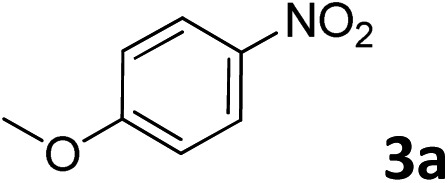	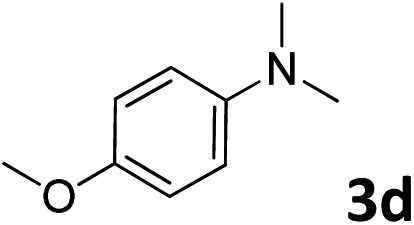	74
3	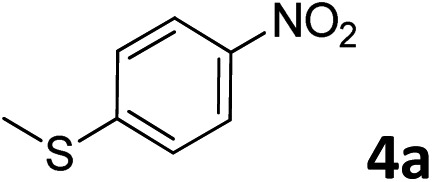	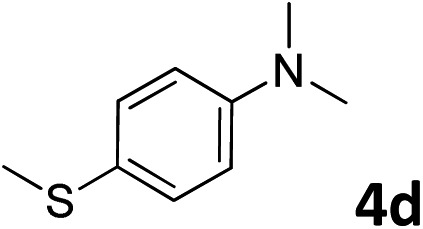	71
4	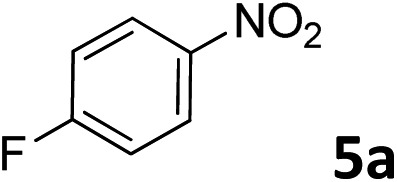	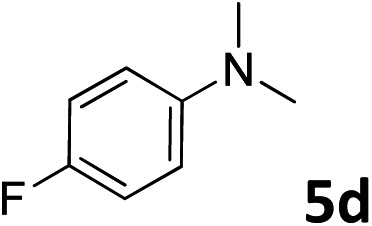	90
5	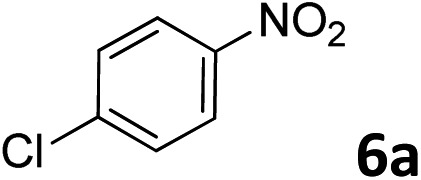	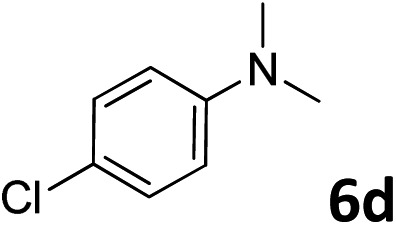	87
6	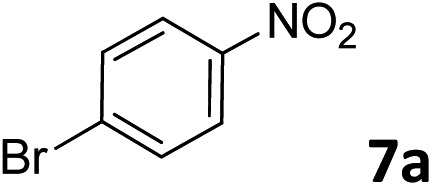	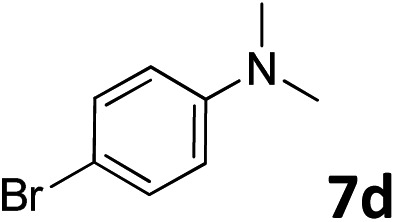	82
7	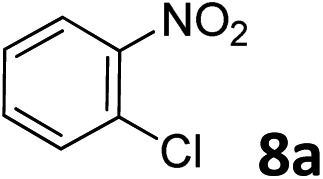	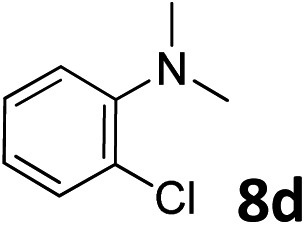	82
8	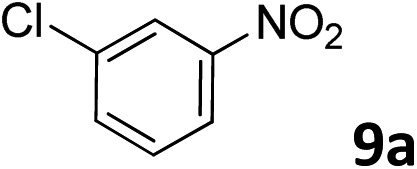	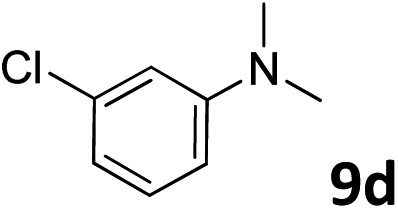	86
9	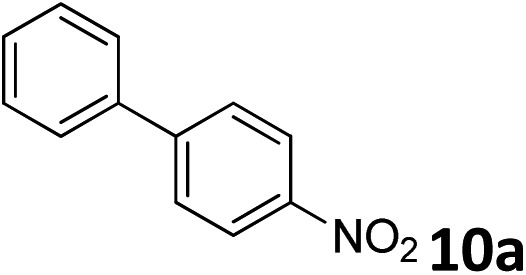	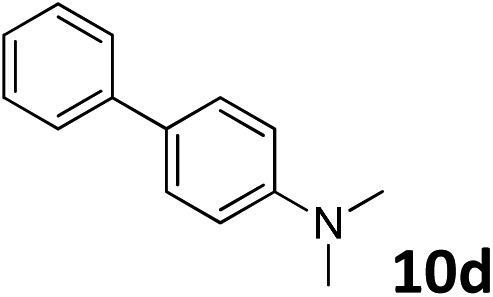	78
10	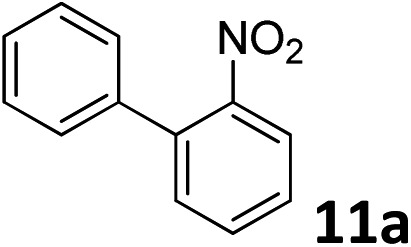	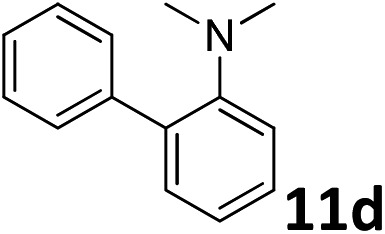	76
11	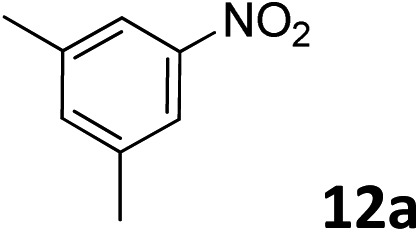	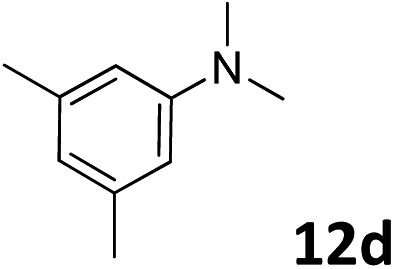	78
12	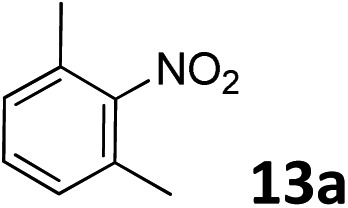	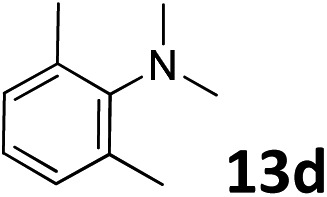	82
13	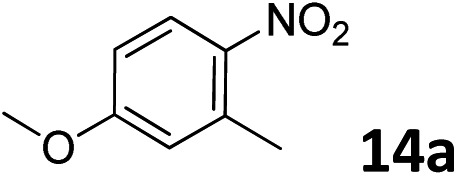	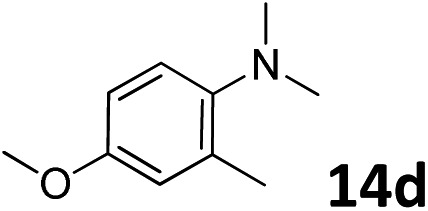	75

^*a*^Reaction conditions: substituted nitrobenzene (1.0 mmol), AMPA (0.06 mmol), CO_2_ (0.1 MPa), electrolyte (30 mL, CO_2_-saturated MeCN containing 0.5 M [Bmim]Tf_2_N), –2.3 V *vs.* Ag/Ag^+^, 30 °C and 10 h.

^*b*^Yields determined by ^1^H NMR spectroscopy.

Some control experiments were conducted in order to obtain some evidence to study the mechanism. Fig. S7† shows that a very small amount of AMPA could promote the reaction to produce *N*,*N*-dimethylaniline efficiently. However, only aniline was formed in the absence of AMPA (Fig. S8†), and aniline could not be further converted even with extending the reaction time (Table S6†). These results indicate that AMPA is necessary and acts as a co-catalyst. To further understand the role of AMPA, NMR analysis was performed on aniline and its mixture with AMPA (Fig. S9†). It was found that the ^1^H signal of N–H in aniline shifted downfield from 4.88 to 5.11 ppm due to mixing with AMPA. The shift of the ^1^H signal of N–H in aniline was also affected by the amount of AMPA and the result was consistent with the reaction result that the yield of the product increased with an increase in the amount of AMPA from 0 to 0.06 mmol (Fig. S7†). From these results, it can be concluded that AMPA as a Brønsted base could activate the proton on aniline to promote the formation of *N*,*N*-dimethylaniline. In this way, the C atom on the intermediates obtained from the CO_2_ electroreduction could promote an easier electrophilic attack to the N atom on aniline, *i.e.* AMPA acted as the co-catalyst to promote the reaction of aniline with the intermediate from the CO_2_ electroreduction to form *N*-phenylformamide or *N*-methyl-*N*-phenylformamide. As a result, the next reaction in which *N*-methyl-*N*-phenylformamide reacted with H^+^ from water to synthesize *N*,*N*-dimethylaniline could proceed successfully. More details will be discussed in the mechanism part below.

**Fig. 2:**
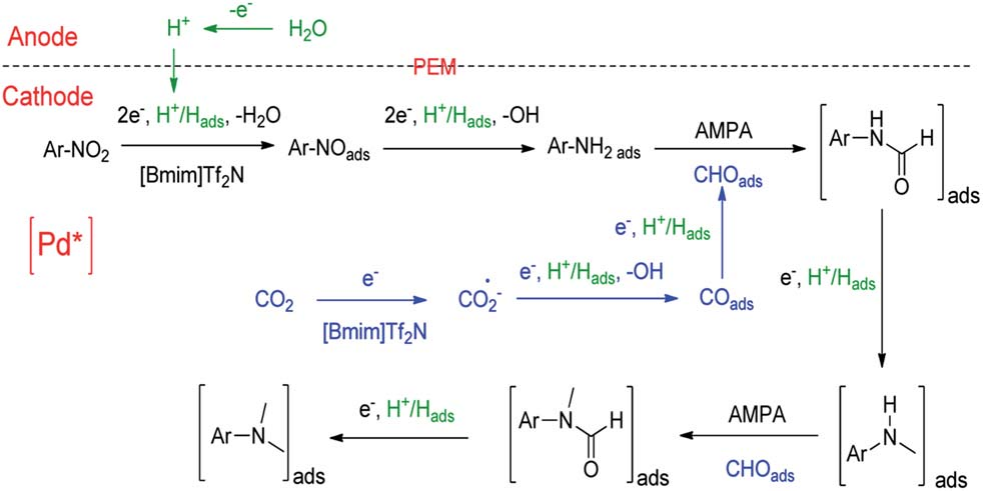
Possible pathway for the electrochemical reaction of nitrobenzene with CO_2_ and water to form *N*,*N*-dimethylaniline over Pd/Co–N/carbon.

On the basis of the experimental results and the related knowledge in the literature, we propose a speculative mechanism for the *N*,*N*-dimethylation of nitrobenzene, which is shown schematically in Fig. 2. Two parallel processes, CO_2_ and nitrobenzene hydrogenation, occur in the cathode compartment in the first step. For CO_2_ activation and reduction, a [Bmim-CO_2_]^+^ complex can form quickly *via* the hydrogen bonding interaction between CO_2_ and the IL cation,^46^ which can reduce the reaction barrier for the electron transfer to CO_2_.^27^ [Bmim-CO_2_]^+^ can be adsorbed on the electrode surface and CO_2_ is reduced to CO_2_˙^–^, which forms CO_ads_ after receiving the second electron. The Pd nanoparticles facilitate these processes.^32^ After accepting an electron and proton, CO_ads_ can be adsorbed on the support, and the pyridinic N as well as the Co–N_*x*_ moieties can further drive CO_ads_ to generate CHO_ads_.^31,39,40^ The next step is the insertion of CHO_ads_ into ArNH_2_Pd* to form adsorbed *N*-phenylformamide in the presence of the Brønsted base, AMPA. ArNH_2_Pd* can be obtained through the pathway of nitrobenzene → nitrosobenzene → *N*-phenylhydroxylamine → aniline on the electrode surface. It is usually observed in the electrochemical reduction of nitrobenzene to aniline.^47^ The adsorbed *N*-phenylformamide can be quickly hydrogenated into *N*-methylaniline. Following this, *N*-methylaniline can be adsorbed onto the electrode surface and reacted with CHO_ads_ to form the adsorbed *N*-methyl-*N*-phenylformamide. Finally, the protonation of the adsorbed *N*-methyl-*N*-phenylformamide leads to the formation of *N*,*N*-dimethylaniline.

To further verify the reaction mechanism proposed, we conducted the experiments using some intermediates in Fig. 2 as the starting reactants, including aniline, *N*-phenylformamide, *N*-methylaniline and *N*-methyl-*N*-phenylformamide (Fig. S10†). 92% *N*,*N*-dimethylaniline, 5% *N*-methylaniline, a small amount of aniline, *N*-phenylformamide and *N*-methyl-*N*-phenylformamide can be detected in reaction (I). In reaction (II), the products contained 93% *N*,*N*-dimethylaniline, about 3% *N*-methylaniline as well as a small amount of *N*-phenylformamide and *N*-methyl-*N*-phenylformamide. The results were similar to those in reaction (I), suggesting that nitrobenzene was first converted to aniline completely in a short time. In reactions (III) and (V), when *N*-phenylformamide or *N*-methyl-*N*-phenylformamide was used as the starting reactant, the desired products *N*-methylaniline or *N*,*N*-dimethylaniline could be obtained. At the same time, most *N*-methylaniline could be transformed to *N*,*N*-dimethylaniline with the aid of AMPA in reaction (IV). In addition, a small amount of *N*-phenylformamide or *N*-methyl-*N*-phenylformamide as the reaction intermediates could also be detected in reactions (III) and (IV). All of this evidence supports the proposed reaction mechanism.

## Conclusions

In conclusion, *N*,*N*-dimethylanilines can be synthesized by the methylation of nitrobenzene (or substituted nitrobenzenes) with CO_2_ and water. A Pd/Co–N/carbon electrode and an electrolyte composed of MeCN, [Bmim]Tf_2_N, and AMPA is a very efficient electrolysis system under ambient conditions. The *N*,*N*-dimethylation of a range of substrates, including nitrobenzene, substituted nitrobenzene and benzonitrile, can proceed smoothly with satisfactory yields. In the reaction, Pd/Co–N/carbon catalyzes the reduction of nitrobenzene to aniline quickly, which is further converted to *N*,*N*-dimethylaniline, catalyzed cooperatively by the electrocatalyst Pd/Co–N/carbon and the thermal catalyst AMPA. We believe that this route has potential application for producing *N*,*N*-dimethylanilines due to some obvious advantages. In addition, the combination of electrocatalysts and thermal catalysts can realize other reactions that cannot be conducted with only electrocatalysts or thermal catalysts.

## Experimental section

### Preparation of Co–ZIF/GO

In this work, GO was prepared by the modified Hummers’ method according to ref. 48. 1.2 mmol of Co(NO_3_)_2_·6H_2_O and 1 mmol of 2-methylimidazole were dissolved in 12 mL and 20 mL of methanol, respectively. A Co(NO_3_)_2_ solution was added to the 2-methylimidazole solution to obtain a clear purple solution under continuous stirring. 10 mL of a GO dispersion (10 mg of GO in 10 mL of methanol/water, v/v, 4 : 1) was then immediately added to the above purple solution. After stirring for 3 h, the precipitate was collected by centrifugation and washing with methanol, followed by drying at 50 °C for 10 h. Co–ZIF/GO was obtained.

### Preparation of Co–N/carbon support

The resulting Co–ZIF/GO precursor was heated in the tube furnace at 10 °C min^–1^ in an argon (Ar) medium to 800 °C and kept at this temperature for 3 h. The composites were then immersed in 2 M HCl aqueous solution for 24 h. The precipitate was collected by centrifugation and washing with water, followed by drying at 80 °C for 24 h. The Co–N/carbon support was obtained, of which the elemental composition is given in Table S1.†

### Preparation of Pd_*x*_/Co–N/carbon catalysts

The Pd/Co–N/carbon catalysts were synthesized with sodium borohydride as a reductive agent, sodium citrate as a stabilizing agent and Co–N/carbon as a support. The sizes of the Pd nanoparticles in the catalysts were controlled by varying the ratio of sodium citrate to PdCl_2_ as well as the reduction temperature.^32^ The average particle size was determined by counting more than 200 particles from the TEM images. Herein, the preparation of Pd_2.2_/Co–N/carbon is described in detail. 0.5 mmol of PdCl_2_ in 0.1 M HCl solution and 4 mmol of sodium citrate were dissolved in 200 mL of water under continuous stirring for 30 min. 200 mg of the as-prepared Co–N/carbon support was then added to the mixture with sonication for 1 h. 50 mL of 0.1 M sodium borohydride aqueous solution was then added to the above suspension dropwise at 0 °C under vigorous stirring to reduce the Pd^2+^. After stirring for 8 h, the black precipitate was collected by centrifugation and washing with water 3 times, and Pd_2.2_/Co–N/carbon was obtained after freeze drying.

The other Pd/Co–N/carbon catalysts were prepared by controlling the reduction temperature and adjusting the ratio of sodium citrate (stabilizing agent) to PdCl_2_. For Pd_3.6_/Co–N/carbon and Pd_4.5_/Co–N/carbon, the temperatures for reducing Pd^2+^ were 25 °C and 60 °C, respectively, and the ratio of sodium citrate to PdCl_2_ was the same as that for preparing Pd_2.2_/Co–N/carbon. For Pd_6.4_/Co–N/carbon and Pd_7.9_/Co–N/carbon, the ratios of sodium citrate to PdCl_2_ were 2 and 0, respectively, and the reduction temperature was 25 °C. Pd_10.2_/Co–N/carbon was obtained by bubbling Pd_3.6_/Co–N/carbon with H_2_ for 12 h in 0.1 M HCl aqueous solution at 25 °C.

### Materials characterization

XPS analysis was performed on the Thermo Scientific ESCALab 250Xi using 200 W monochromatic Al Kα radiation. The 500 μm X-ray spot was used. The base pressure in the analysis chamber was about 3 × 10^–10^ mbar. Typically, the hydrocarbon C 1s line at 284.8 eV from the adventitious carbon was used for energy referencing. The actual loading of Pd in the catalysts was determined by ICP-OES (Vista-MPX). The N_2_ adsorption/desorption isotherms of the Co–N/carbon nanosheets were determined using a Quadrasorb SI-MP system. The microstructures of the catalysts were studied using JEOL-2100F HR-TEM operated at 200 kV.

### Preparation of the electrode and electrochemical reaction

To prepare an electrode, the corresponding Pd_*x*_/Co–N/carbon catalyst was suspended in acetone with Nafion D-521 dispersion to form a homogeneous ink with the aid of ultrasound, which was spread onto carbon paper (CP: 1 × 1 cm^–2^) to obtain the working electrodes. The loading of the catalyst was 2.0 ± 0.1 mg cm^–2^. Before the experiment, all of the auxiliary electrodes were sonicated in acetone for 3 min and then washed with water and ethanol, followed by drying under a N_2_ atmosphere.

An electrochemical workstation (CHI 6081E, Shanghai CH Instruments Co., China) was used in all of the experiments. The electrolysis experiments were conducted in a typical H-type cell that was similar to that used in previous works,^28,29^ and is schematically shown in Fig. S2.† It consisted of a cathode (working electrode), an anode (platinum gauze auxiliary electrode), and an Ag/Ag^+^ reference electrode. The cathode and anode compartments were separated by a Nafion 117 proton exchange membrane. A 0.5 M H_2_SO_4_ aqueous solution was used as the anodic electrolyte. H^+^ can be transferred from the anode compartment to the cathode compartment through the Nafion 117 proton exchange membrane, which is the hydrogen source. Under continuous stirring, CO_2_ was bubbled through the catholyte (2 mL min^–1^) for 30 min before electrolysis. The electrolysis experiments were then carried out with CO_2_ bubbling (2 mL min^–1^). In a typical reaction procedure, 1.0 mmol of nitrobenzene and 0.06 mmol of AMPA were added to 30 mL of the electrolyte (CO_2_-saturated MeCN containing 0.5 M [Bmim]Tf_2_N). The reaction was performed at 30 °C for the desired time under magnetic stirring. After the reaction, MeCN in the system was removed by rotary evaporation and the IL was extracted using ether. The isolated product was obtained and purified by column chromatography using petroleum ether/ethyl acetate (150 : 1).

### Product analysis

The gaseous product of the electrochemical experiments was collected and analyzed by gas chromatography (GC, HP 4890D), which was equipped with FID and TCD detectors, using helium as the internal standard. For the methylation of nitrobenzene, the liquid product was analyzed using ^1^H NMR (Bruker Avance III 400 HD spectrometer) in chloroform-d, with TMS as an internal standard.
