# Supplementary material for: N,N-Dimethylation of nitrobenzenes with CO2 and water by electrocatalysis
Source: Chem Sci. 2017 Jun 7;8(8):5669–74. doi: 10.1039/c7sc01058c (PMC5621054; doi:10.1039/c7sc01058c)
Supplement: Supplementary file 1 [file SC-008-C7SC01058C-s001.pdf]

Electronic Supplementary Material (ESI) for Chemical Science.
This journal is © The Royal Society of Chemistry 2017

Electronic Supplementary Information

N,N-Dimethylation of Nitrobenzenes with CO_2 and Water by Electrocatalysis

Xiaofu Sun, Qinggong Zhu, Jiayin Hu, Xinchen Kang, Jun Ma, Huizhen Liu, and Buxing Han*

Experimental Section

Materials

1-Butyl-3-methylimidazolium bis(trifluoromethylsulfonyl)imide ([Bmim]Tf_2N, purity > 99%), 1-butyl-3-methylimidazolium tetrafluoroborate ([Bmim]BF_4, purity > 99%), 1-butyl-3-methylimidazolium hexafluorophosphate ([Bmim]PF_6, purity > 99%), 1-butyl-3-methylimidazolium perchlorate ([Bmim]ClO_4, purity > 99%), 1-butyl-3-methylimidazolium nitrate ([Bmim]NO_3, purity > 99%), 1-butyl-3-methylimidazolium dihydrogen phosphate ([Bmim]H_2PO_4, purity > 99%), and 1-butyl-3-methylimidazolium trifluoromethansulfonate ([Bmim]TfO, purity > 99%) were provided by the Centre of Green Chemistry and Catalysis, Lanzhou Institute of Chemical Physics, Chinese Academy of Sciences. Nitrobenzene (99 %), aniline (99.5 %) and N-methylaniline (99 %), palladium (II) chloride (Pd ≥ 59.0 %), cobalt nitrate hexahydrate (99 %) were purchased from Sinopharm Chem. Reagent Co. Ltd. 4-Nitrothioanisole (98 %), 1-bromo-4-nitrobenzene (98 %), 5-nitro-m-xylene (99 %), 1-amino-methylphosphonic acid (99 %), benzonitrile (99 %), 4-methoxybenzonitrile (99 %), sodium borohydride (98 %), Nafion N-117 membrane (0.180 mm thick, ≥ 0.90 meg/g exchange capacity), Nafion D-521 dispersion (5 % w/w in water and 1-propanol, ≥ 0.92 meg/g exchange capacity) and Toray Carbon Paper (CP, TGP-H-60, 19×19 cm) were obtained from Alfa Aesar China Co., Ltd. 4-Chloronitrobenzene (98 %), 2-chloronitrobenzene (99 %), 1-chloro-3-nitrobenzene (99 %), 2-nitrobiphenyl (98 %), 2,6-dimethylnitrobenzene (97 %) and p-tolunitrile (98 %) were provided by Tokyo Chemical Industry Co., Ltd. 4-Nitrotoluene (99 %), 4-nitroanisole (99 %) and 2-methylimidazole (99 %) were purchased from Acros Organics. 4-Fluoronitrobenzene (99 %), 4-fluorobenzonitrile (98 %) and 4-chlorobenzonitrile (98 %) were obtained from Inno-chem Beijing Co., Ltd. 4-Nitrobiphenyl (95 %) and sodium citrate (99 %) were purchased from J&K Scientific Ltd. 3-Methyl-4-nitroanisole (98 %) was purchased from Adamas Reagent Co., Ltd.

^{1}H, ^{13}C NMR and MS data of the isolated compounds

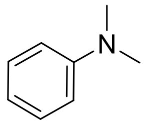

1d: Yield: 86 %. ^{1}H NMR (400 MHz, $CDCl_3$): δ 7.22 (t, *J* = 8.4 Hz, 2H), 6.60-6.77 (m, 3H), 2.91 (s, 6H); ^{13}C NMR (100 MHz, $CDCl_3$): δ 150.76, 129.14, 116.74, 112.48, 40.67. MS (EI): *m*/*z* (rel. int.) 121.

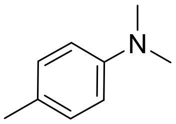

2d: Yield: 70 %. ^{1}H NMR (400 MHz, $CDCl_3$): δ 7.08 (t, *J* = 10.3 Hz, 2H), 6.72 (t, *J* = 5.7 Hz, 2H), 2.90 (s, 6H), 2.25 (s, 3H); ^{13}C NMR (100 MHz, $CDCl_3$): δ 148.88, 129.65, 126.23, 113.70, 41.12, 20.33. MS (EI): *m*/*z* (rel. int.) 135.

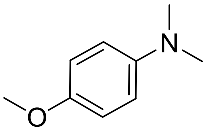

3d: Yield: 66 %. ^{1}H NMR (400 MHz, $CDCl_3$): δ 6.85-6.87 (m, 2H), 6.76-6.80 (m, 2H), 3.78 (s, 3H), 2.89 (s, 6H); ^{13}C NMR (100 MHz, $CDCl_3$): δ 151.98, 145.69, 114.90, 114.58, 55.71, 41.81. MS (EI): *m*/*z* (rel. int.) 151.

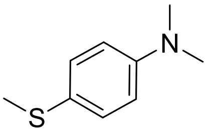

4d: Yield: 67 %. ^{1}H NMR (400 MHz, $CDCl_3$): δ 7.14-7.18 (m, 2H), 6.57-6.62 (m, 2H), 2.90 (s, 6H), 2.39 (s, 3H); ^{13}C NMR (100 MHz, $CDCl_3$): δ 145.21, 131.34, 130.80, 125.61, 41.30, 18.80. MS (EI): *m*/*z* (rel. int.) 167.

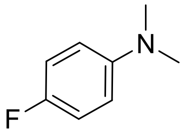

5d: Yield: 85 %. ^{1}H NMR (400 MHz, $CDCl_3$): δ 6.88-7.13 (m, 2H), 6.64-6.75 (m, 2H), 2.90 (s, 6H); ^{13}C NMR (100 MHz, $CDCl_3$): δ 156.85, 154.92, 147.66, 115.49, 114.02 (d, *J* = 7.4 Hz), 41.40. MS (EI): *m*/*z* (rel. int.) 139.

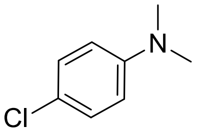

6d: Yield: 81 %. ^{1}H NMR (400 MHz, $CDCl_3$): δ 7.16 (d, *J* = 9.2 Hz, 2H), 6.62 (d, *J* = 8.8 Hz, 2H), 2.91 (s, 6H); ^{13}C NMR (100 MHz, $CDCl_3$): δ 149.21, 128.82, 121.50, 113.69, 40.66. MS (EI): *m*/*z* (rel. int.) 155.

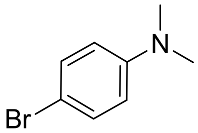

7d: Yield: 80 %. ^{1}H NMR (400 MHz, $CDCl_3$): δ 7.28 (d, *J* = 9.2 Hz, 2H), 6.51-6.65 (m, 2H), 2.91 (s, 6H); ^{13}C NMR (100 MHz, $CDCl_3$): δ 149.52, 131.70, 114.16, 108.60, 40.58. MS (EI): *m*/*z* (rel. int.) 200.

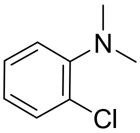

8d: Yield: 76 %. ^{1}H NMR (400 MHz, $CDCl_3$): δ 7.33-7.35 (m, 1H), 7.15-7.24 (m, 1H), 7.05-7.07 (m, 1H), 6.91-6.96 (m, 1H), 2.81 (s, 6H); ^{13}C NMR (100 MHz, $CDCl_3$): δ 150.36, 144.92, 130.68, 127.36, 123.09, 119.96, 43.26. MS (EI): *m*/*z* (rel. int.) 155.

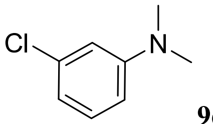

9d: Yield: 75 %. ^{1}H NMR (400 MHz, $CDCl_3$): δ 7.13 (m, 1H), 6.65-6.71 (m, 2H), 6.52-6.62 (m, 1H), 2.92 (s, 6H); ^{13}C NMR (100 MHz, $CDCl_3$): δ 151.53, 135.01, 129.96, 116.24, 112.27, 110.54, 40.37. MS (EI): *m*/*z* (rel. int.) 155.

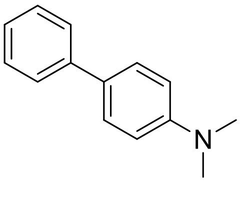

10d: Yield: 70 %. ^{1}H NMR (400 MHz, $CDCl_3$): δ 7.53 (d, *J* = 7.4 Hz, 2H), 7.40 (dd, *J* = 13.5, 8.0 Hz, 4H), 7.28 (m, 1H), 6.75 (d, *J* = 8.3 Hz, 2H), 2.96 (s, 6H); ^{13}C NMR (100 MHz, $CDCl_3$): δ 145.84, 141.19, 131.63, 128.66, 128.02, 126.33 (d, *J* = 15.5 Hz), 115.41, 41.26. MS (EI): *m*/*z* (rel. int.) 197.

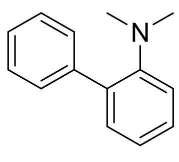

11d: Yield: 67 %. ^{1}H NMR (400 MHz, $CDCl_3$): δ 7.40-7.48 (m, 3H), 7.31-7.36 (m, 2H), 7.08-7.18 (m, 2H), 6.72-6.85 (m, 2H), 2,88 (s, 6H); ^{13}C NMR (100 MHz, $CDCl_3$): δ 143.48, 139.60, 130.45, 129.11, 128.80, 128.49, 127.72, 127.16, 118.68, 115.63, 42.33. MS (EI): *m*/*z* (rel. int.) 197.

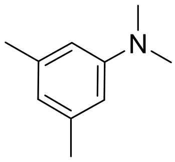

12d: Yield: 71 %. ^{1}H NMR (400 MHz, $CDCl_3$): δ 6.37-6.41 (m, 1H), 6.31 (s, 2H), 2.95 (s, 6H), 2.21 (s, 6H); ^{13}C NMR (100 MHz, $CDCl_3$): δ 146.39, 139.01, 120.52, 113.14, 41.86, 21.33. MS (EI): *m*/*z* (rel. int.) 149.

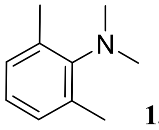

13d: Yield: 74 %. ^{1}H NMR (400 MHz, $CDCl_3$): δ 6.93 (d, J = 7.4 Hz, 2H), 6.63 (t, J = 7.5 Hz, 1H), 2.94 (s, 6H), 2.16 (s, 6H); ^{13}C NMR (100 MHz, $CDCl_3$): δ 142.76, 128.31, 121.74, 118.32, 41.30, 17.63. MS (EI): *m*/*z* (rel. int.) 149.

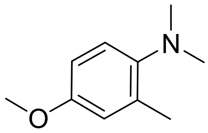

14d: Yield: 70 %. ^{1}H NMR (400 MHz, $CDCl_3$): δ 6.78 (m, 1H), 6.58-6.60 (m, 1H), 6.48-6.52 (m, 1H), 3.86 (s, 3H), 2.94 (s, 6H), 2.12 (s, 3H); ^{13}C NMR (100 MHz, $CDCl_3$): δ 150.25, 140.62, 129.14, 121.55, 113.26, 108.66, 53.28, 42.56, 19.24. MS (EI): *m*/*z* (rel. int.) 165.

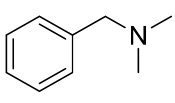

15d: Yield: 58 %. ^{1}H NMR (400 MHz, $CDCl_3$): δ 7.15-7.25 (m, 5H), 3.41 (s, 2H), 2.23 (s, 6H); ^{13}C NMR (100 MHz, $CDCl_3$): δ 138.90, 129.05, 128.16, 127.03, 64.45, 45.38. MS (EI): *m*/*z* (rel. int.) 135.

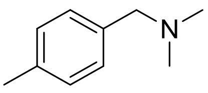 **16d**: Yield: 47 %. ^{1}H NMR (400 MHz, $CDCl_3$): δ 7.02-7.08 (m, 4H), 3.58 (s, 2H), 2.27 (s, 3H), 2.18 (s, 6H); ^{13}C NMR (100 MHz, $CDCl_3$): δ 136.27, 135.62, 129.75, 128.10, 67.35, 46.69, 21.80. MS (EI): *m/z* (rel. int.) 149.

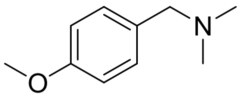

17d: Yield: 40 %. ^{1}H NMR (400 MHz, $CDCl_3$): δ 7.20-7.24 (m, 2H), 6.85-6.87 (m, 2H), 3.78 (s, 3H), 3.38 (s, 2H), 2.25 (s, 6H); ^{13}C NMR (100 MHz, $CDCl_3$): δ 158.37, 135.38, 128.84, 114.26, 63.62, 55.16, 45.72. MS (EI): *m/z* (rel. int.) 165.

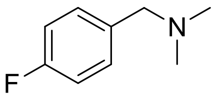

18d: Yield: 57 %. ^{1}H NMR (400 MHz, $CDCl_3$): δ 7.18-7.24 (m, 2H), 7.02-7.07 (m, 2H), 3.58 (s, 2H), 2.20 (s, 6H); ^{13}C NMR (100 MHz, $CDCl_3$): δ 158.33, 134.68, 130.72, 116.74, 67.26, 45.02. MS (EI): *m/z* (rel. int.) 153.

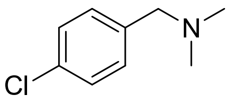

19d: Yield: 45 %. ^{1}H NMR (400 MHz, $CDCl_3$): δ 7.28-7.32 (m, 2H), 7.21-7.25 (m, 2H), 3.60 (s, 2H), 2.18 (s, 6H); ^{13}C NMR (100 MHz, $CDCl_3$): δ 138.56, 133.38, 129.70, 117.93, 67.46, 45.28. MS (EI): *m/z* (rel. int.) 169.

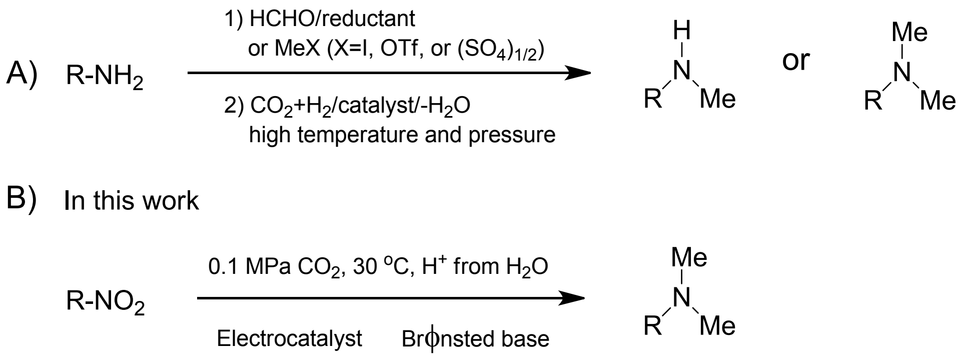

Fig. S1. The comparison between reported methods [S1-S7] and route of this work for the synthesis of N,N-dimethylanilines.

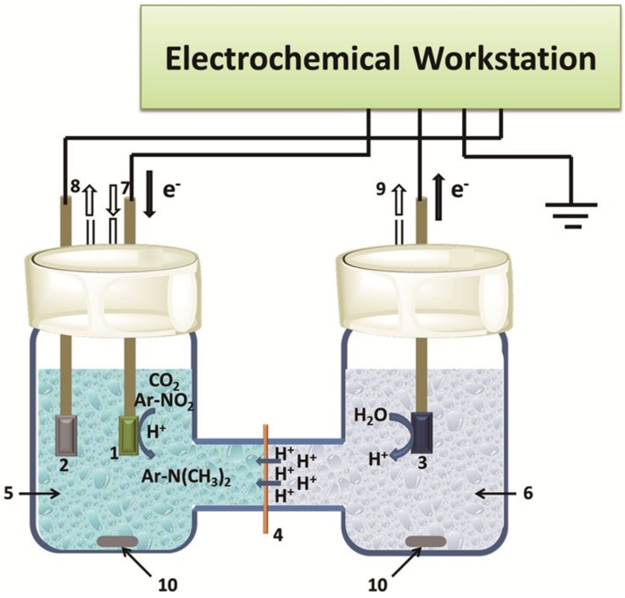

Fig. S2. The schematic diagram of the electrolysis device (H-type cell) and principle of the reaction.

1. working electrode, 2. reference electrode, 3. auxiliary electrode, 4. Nafion 117 membrane, 5. electrolyte, 6. 0.5 mol/L H_2SO_4 aqueous solution, 7. CO_2 inlet, 8. gas product outlet, 9. O_2 outlet, 10. magnetic stirrer.

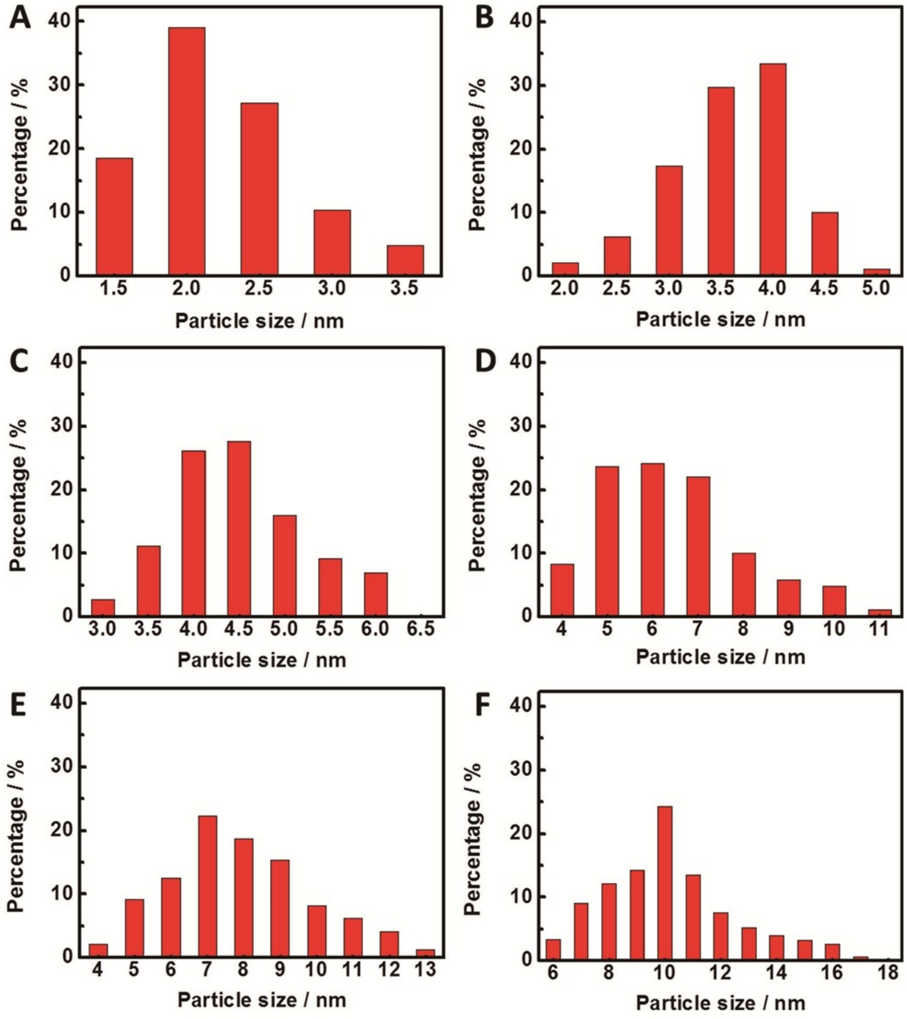

Fig. S3. The size distributions of Pd particles in different catalysts. (A) $Pd_{2.2}$/Co-N/carbon, (B) $Pd_{3.6}$/Co-N/carbon, (C) $Pd_{4.5}$/Co-N/carbon, (D) $Pd_{6.4}$/Co-N/carbon, (E) $Pd_{7.9}$/Co-N/carbon, and (F) $Pd_{10.2}$/Co-N/carbon.

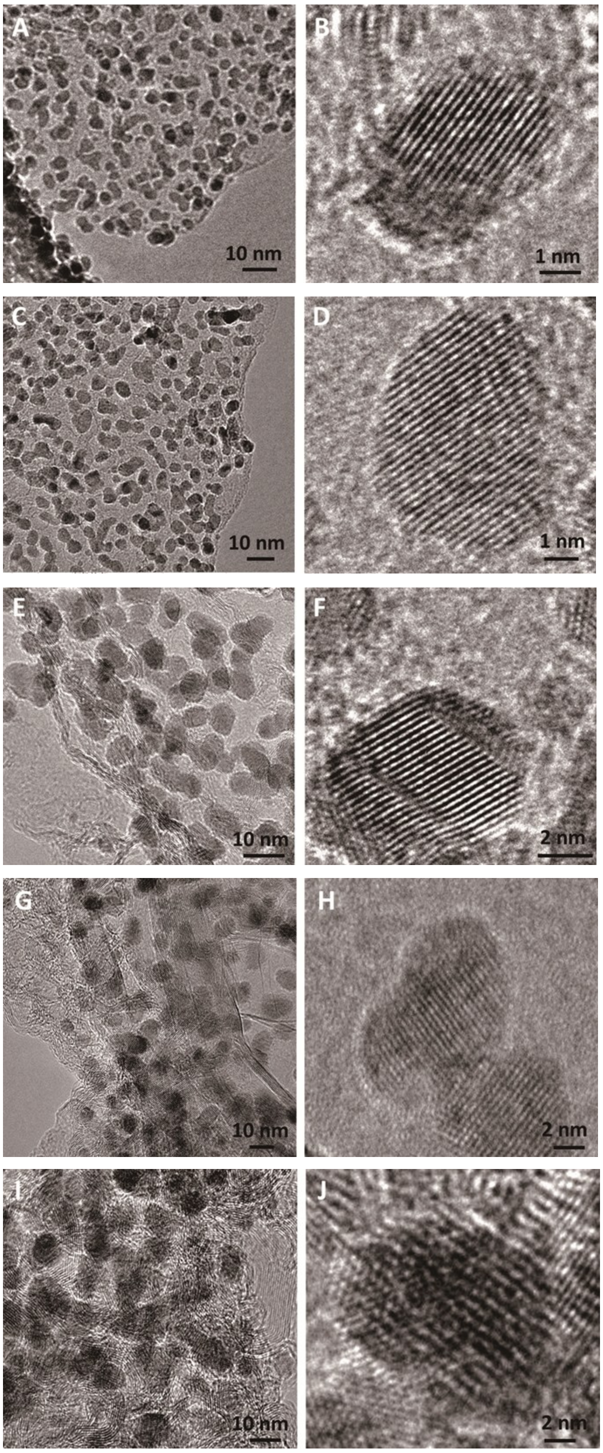

Fig. S4. TEM and HR-TEM images of different catalysts. (A, B) $Pd_{3.6}$/Co-N/carbon, (C, D) $Pd_{4.5}$/Co-N/carbon, (E, F) $Pd_{6.4}$/Co-N/carbon, (G, H) $Pd_{7.9}$/Co-N/carbon, and (I, J) $Pd_{10.2}$/Co-N/carbon.

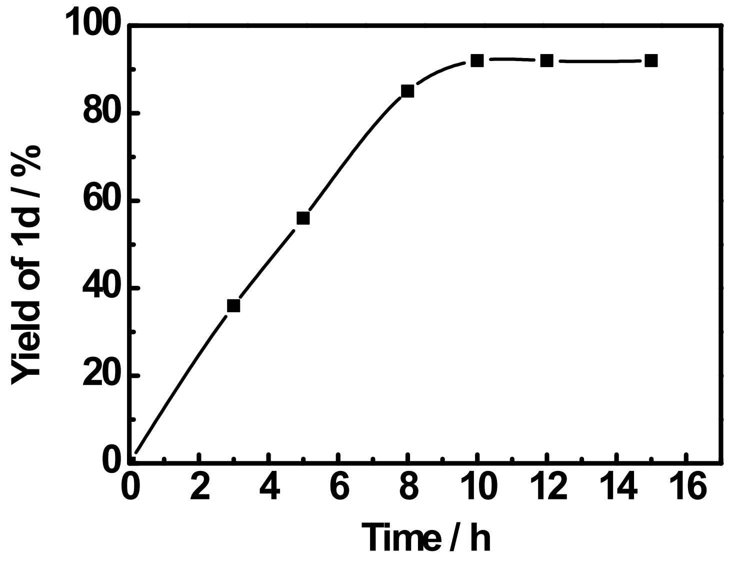

Fig. S5. The yield of *N,N*-dimethylaniline **1d** versus reaction time as the reaction condition of Table 1, entries 5, 7-10.

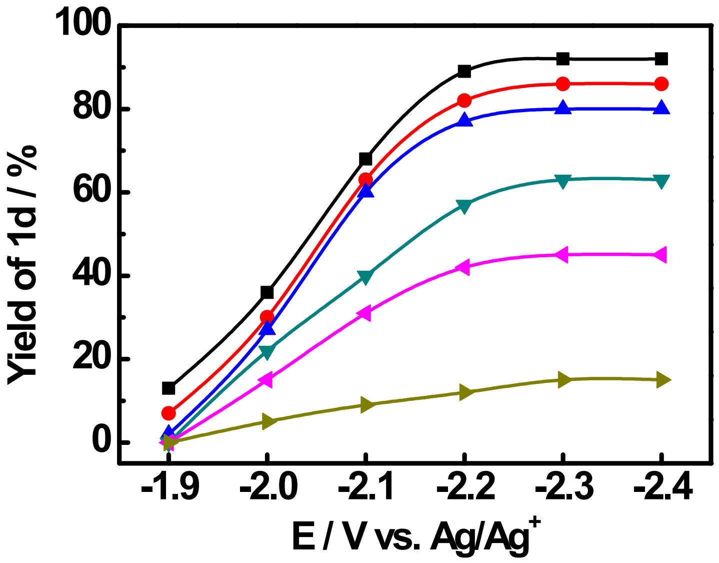

Fig. S6. The yields of N,N-dimethylaniline **1d** from the electrochemical conversion of nitrobenzene **1a** over Pd/Co-N/carbon catalysts with different Pd particle sizes at various potentials. From top to bottom: $Pd_{2.2}$/Co-N/carbon, $Pd_{3.6}$/Co-N/carbon, $Pd_{4.5}$/Co-N/carbon, $Pd_{6.4}$/Co-N/carbon, $Pd_{7.9}$/Co-N/carbon, and $Pd_{10.2}$/Co-N/carbon.

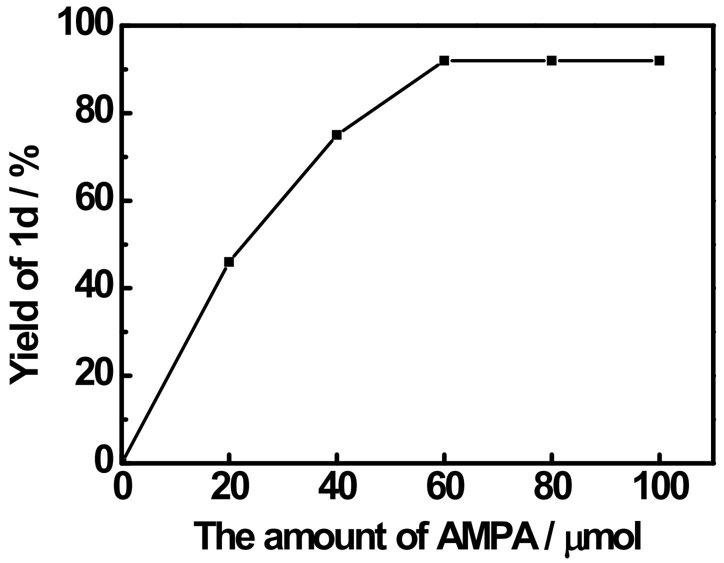

Fig. S7. The yield of *N,N*-dimethylaniline **1d** versus the moles of AMPA (in 30 mL electrolyte) at the reaction condition of Table 1, entry 5.

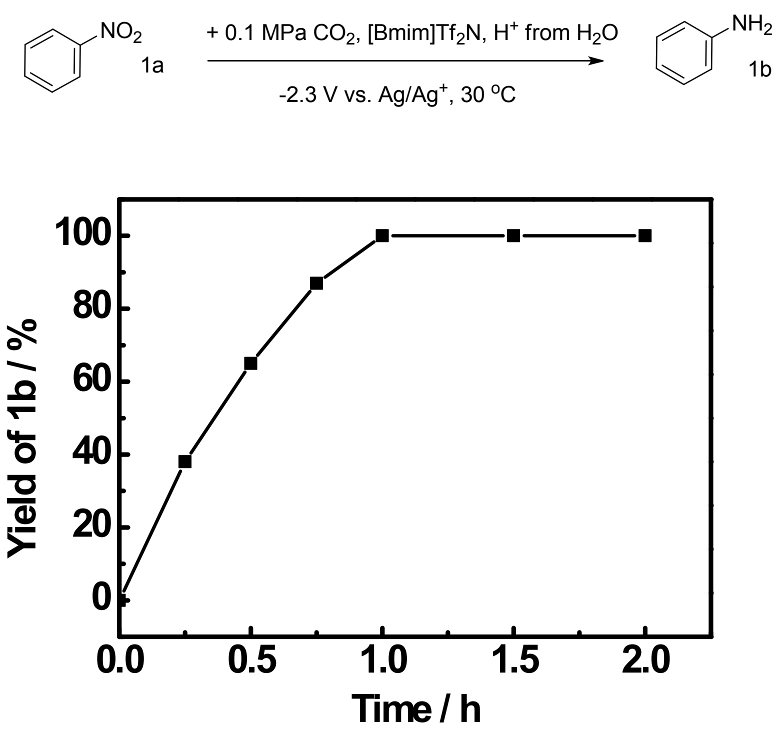

Fig. S8. The yield of aniline **1b** from the methylation of nitrobenzene **1a** versus reaction time without AMPA

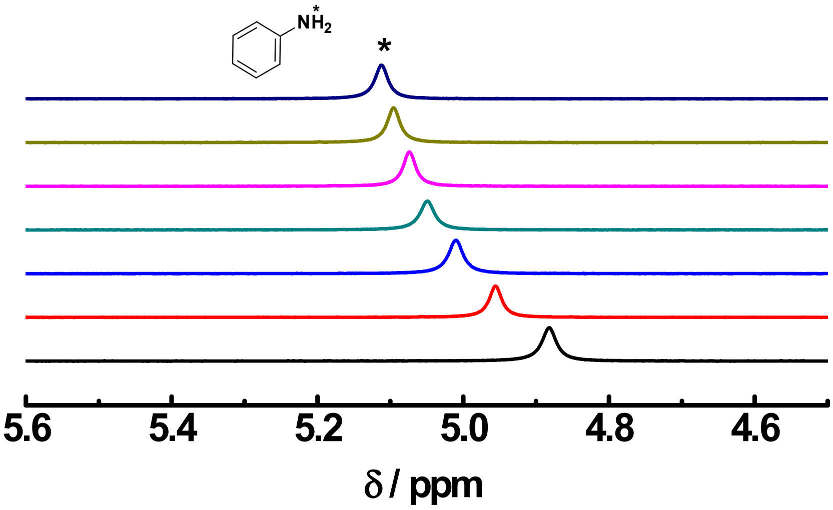

Fig. S9. ^{1}H NMR spectra of aniline and its mixture with different amount of AMPA in DMSO-d_6. Each sample was composed of 0.5 mmol of aniline, 0.6 mL of DMSO-d_6 and a certain amount of AMPA (from botoom to top: 0, 0.005, 0.01, 0.015, 0.02, 0.025 and 0.03 mmol).

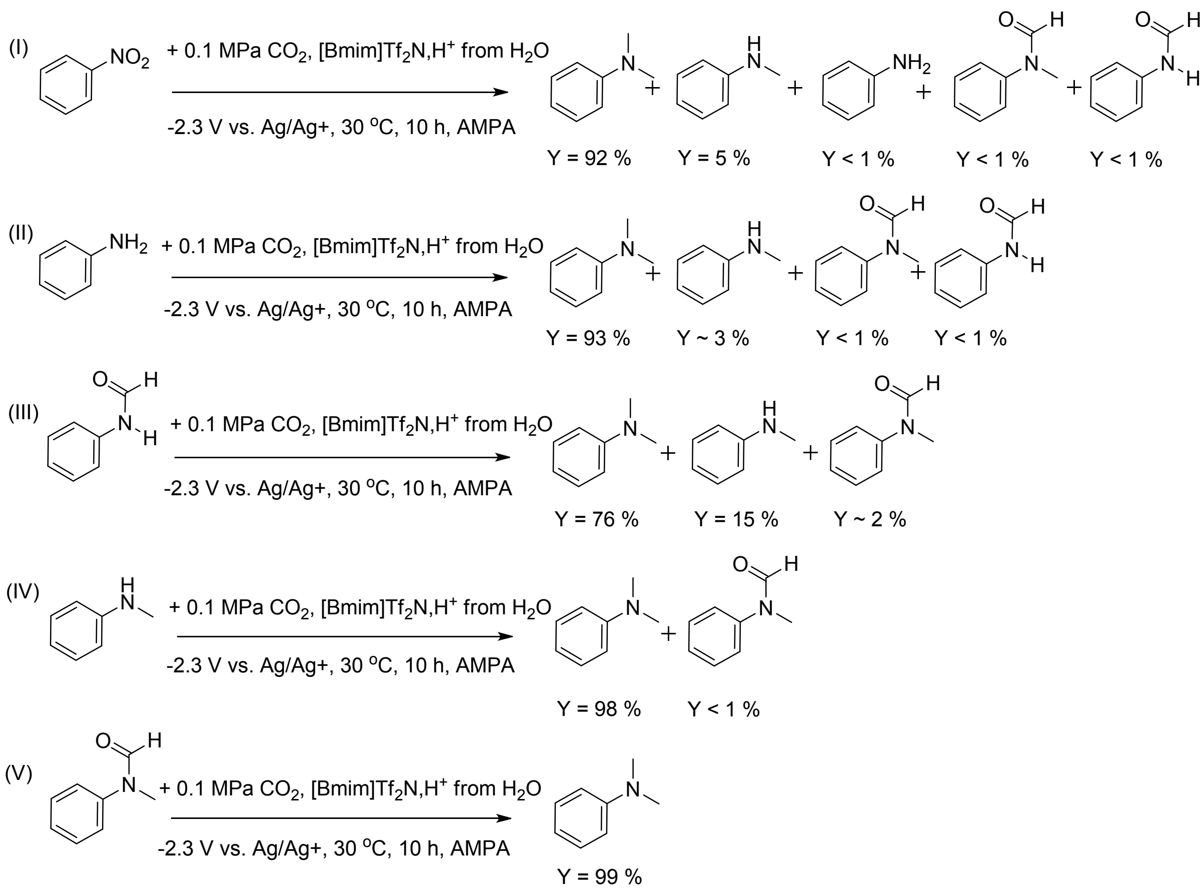

Fig. S10. Reaction mechanism exploration of the electrochemical N-methylation reaction over $Pd_{2.2}$/Co-N/carbon. Reaction conditions: reactant (1.0 mmol), AMPA (0.06 mmol), CO_2 (0.1 MPa), electrolyte (30 mL, CO_2-saturated MeCN containing 0.5 M [Bmim]Tf_2N), -2.3 V vs Ag/Ag^+, 30 ^oC, 10 h.

Table S1. The composition of Co-N/carbon support determined by XPS.

Co / atomic %	N / atomic %	C / atomic %	O / atomic %
16.20	3.16	34.72	45.18

Table S2. The nominal and actual loadings of Pd and average particle size of Pd in different catalysts.

	Pd $loading_{nominal}$ /wt%	Pd $loading_{actual}$ /wt%[a]	Average size of Pd/nm[b]
$Pd_{2.2}$/Co-N/carbon	20	18.3	2.2
$Pd_{3.6}$/Co-N/carbon	20	18.6	3.6
$Pd_{4.5}$/Co-N/carbon	20	18.4	4.5
$Pd_{6.4}$/Co-N/carbon	20	18.8	6.4
$Pd_{7.9}$/Co-N/carbon	20	17.7	7.9
$Pd_{10.2}$/Co-N/carbon	20	18.5	10.2

[a] The values were detected by ICP-AES. [b] The average particle size was obtained by counting more than 200 particles in corresponding TEM images.

Table S3. Methylation of nitrobenzene with CO_2 and water over $Pd_{2.2}$/Co-N/carbon at different temperature.[a]

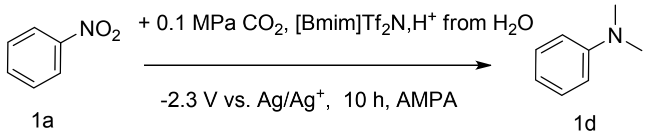

Entry	Temperature / °C	Yield / %[b]
1	20	65
2	30	92
3	40	81
4	50	72
5	60	58

[a] Reaction conditions: nitrobenzene (1.0 mmol), AMPA (0.06 mmol), CO_2 (0.1 MPa), electrolyte (30 mL, CO_2-saturated MeCN containing 0.5 M [Bmim]Tf_2N); [b] Yield determined by ^1H NMR spectroscopy.

Table S4. Methylation of nitrobenzene with CO_2 and water in different solvents.[a]

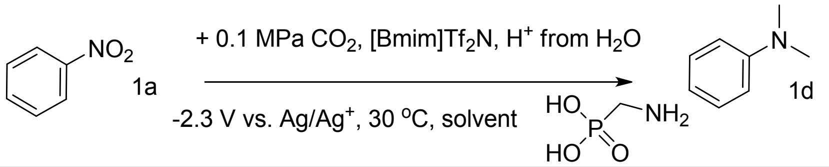

Entry	Solvent[b]	Yield / %[c]
1	MeCN	92
2	DMSO	65
3	DMF	43
4	$MeNO_2$	11
5	1,4-dioxane	8

[a] Reaction conditions: nitrobenzene (1.0 mmol), AMPA (0.06 mmol), CO_2 (0.1 MPa), reaction time (10 h); [b] Electrolyte (30 mL) is CO_2-saturated solvent containing 0.5 M [Bmim]Tf_2N; [c] Yield determined by ^1H NMR spectroscopy.

Table S5. $Pd_{2.2}$/Co-N/carbon-catalyzed methylation of benzonitrile and its derivatives with CO_2.[a]

Entry	Substrates	Products	Yield / %[b]
1	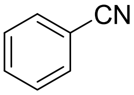 15a	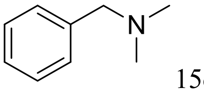 15d	65
2	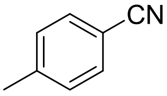 16a	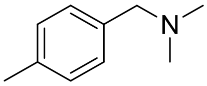 16d	52
3	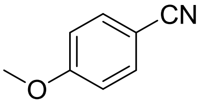 17a	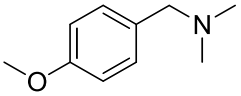 17d	46
4	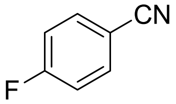 18a	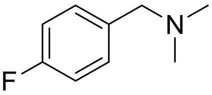 18d	61
5	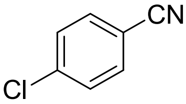 19a	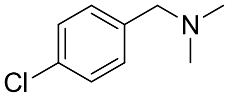 19d	52

[a] Reaction conditions: substituted benzonitrile (1.0 mmol), AMPA (0.06 mmol), CO_2 (0.1 MPa), electrolyte (30 mL, CO_2-saturated MeCN containing 0.5 M [Bmim]Tf_2N), -2.3 V vs Ag/Ag^+, 30 ^oC, 10 h; [b] Yield determined by ^1H NMR spectroscopy.

Table S6. $Pd_{2.2}$/Co-N/carbon-catalyzed methylation of nitrobenzene with CO_2 in absence of 1-amino-methylphosphonic acid (AMPA).[a]

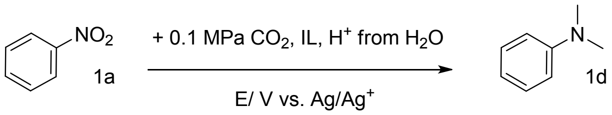

Entry	Electrolyte[b]	E / V[c]	T / °C	t / h	Yield / %[d]
1	[Bmim]Tf_2N	-1.9	30	10	0
2	[Bmim]Tf_2N	-2.0	30	10	0
3	[Bmim]Tf_2N	-2.1	30	10	0
4	[Bmim]Tf_2N	-2.2	30	10	0
5	[Bmim]Tf_2N	-2.3	30	10	0
6	[Bmim]Tf_2N	-2.4	30	10	0
7	[Bmim]Tf_2N	-2.3	20	10	0
8	[Bmim]Tf_2N	-2.3	40	10	0
9	[Bmim]Tf_2N	-2.3	60	10	0
10	[Bmim]Tf_2N	-2.3	30	12	0
11	[Bmim]Tf_2N	-2.3	30	20	0

[a] Reaction conditions: nitrobenzene (1.0 mmol), CO_2 (0.1 MPa); [b] Electrolyte (30 mL) is CO_2-saturated MeCN containing 0.5 M IL; [c] All potentials are reported with respect to Ag/Ag^+. [d] Yield determined by ^1H NMR spectroscopy.

References

S1. K. Beydoun, T. vom Stein, J. Klankermayer and W. Leitner, *Angew. Chem. Int. Ed.*, 2013, **52**, 9554.

S2. Y. Li, X. Fang, K. Junge and M. Beller, *Angew. Chem. Int. Ed.*, 2013, **52**, 9568.

S3. Y. Li, I. Sorribes, T. Yan, K. Junge and M. Beller, *Angew. Chem. Int. Ed.*, 2013, **52**, 12156.

S4. I. Sorribes, J. R. Cabrero-Antonino, C. Vicent, K. Junge and M. Beller, *J. Am. Chem. Soc.*, 2015, **137**, 13580.

S5. E. Blondiaux, J. Pouessel and T. Cantat, *Angew. Chem. Int. Ed.*, 2014, **53**, 12186.

S6. X. Cui, X. Dai, Y. Zhang, Y. Deng and F. Shi, *Chem. Sci.*, 2014, **5**, 649.

S7. O. Jacquet, X. Frogneux, C. Das Neves Gomes and T. Cantat, *Chem. Sci.*, 2013, **4**, 2127.

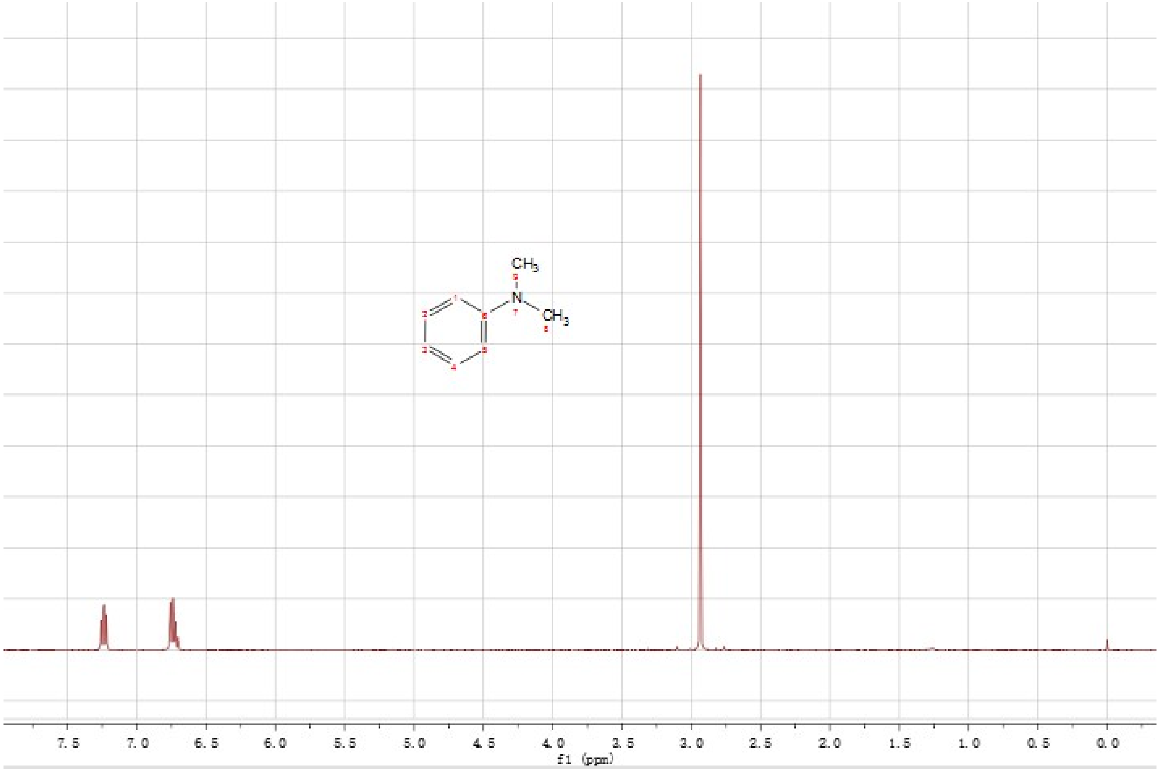
CH3
N
CH3
7.5 7.0 6.5 6.0 5.5 5.0 4.5 4.0 3.5 3.0 2.5 2.0 1.5 1.0 0.5 0.0
f1 (ppm)

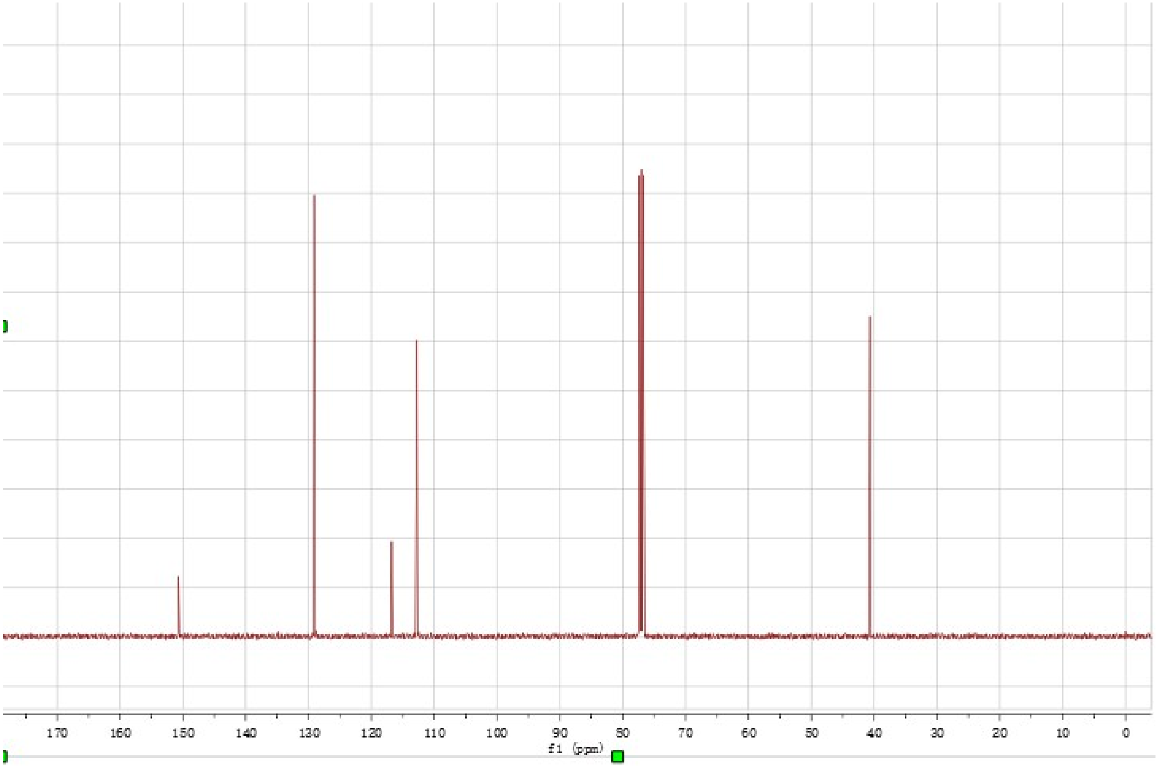
170 160 150 140 130 120 110 100 90 80 70 60 50 40 30 20 10 0
f1 (ppm)

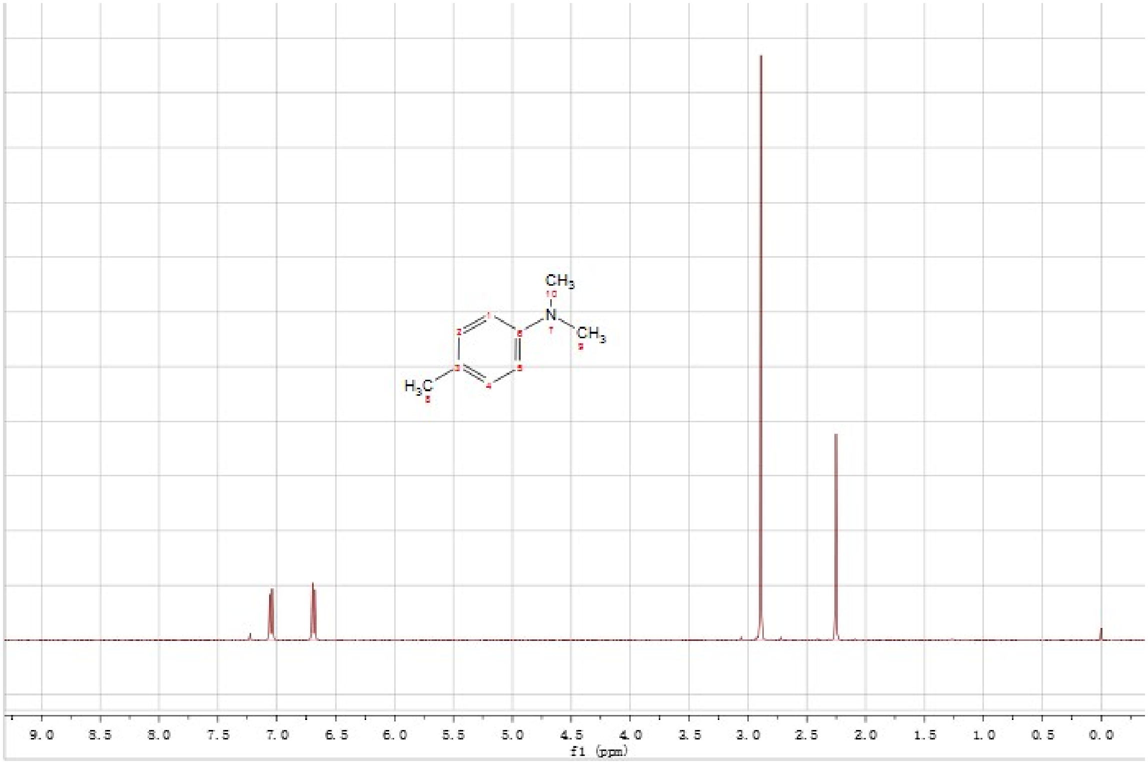
CH3
N
CH3
H3C
9.0 8.5 8.0 7.5 7.0 6.5 6.0 5.5 5.0 4.5 4.0 3.5 3.0 2.5 2.0 1.5 1.0 0.5 0.0
f1 (ppm)

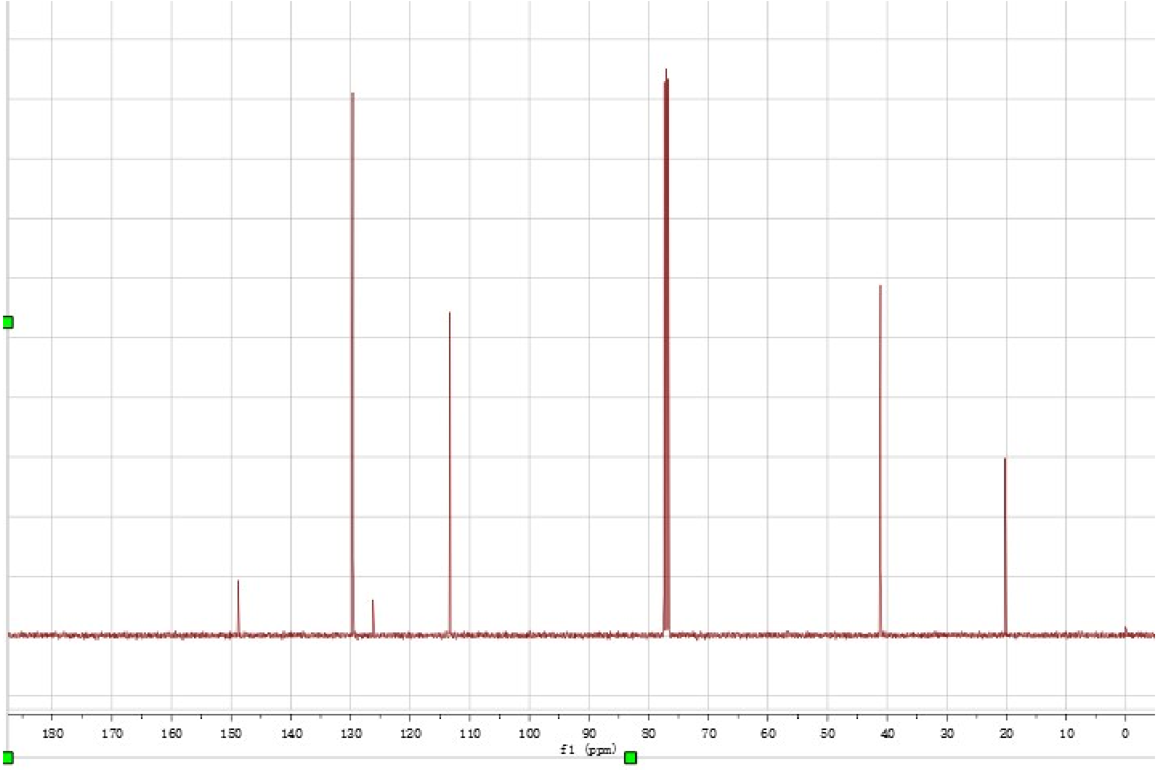
180 170 160 150 140 130 120 110 100 90 80 70 60 50 40 30 20 10 0
f1 (ppm)

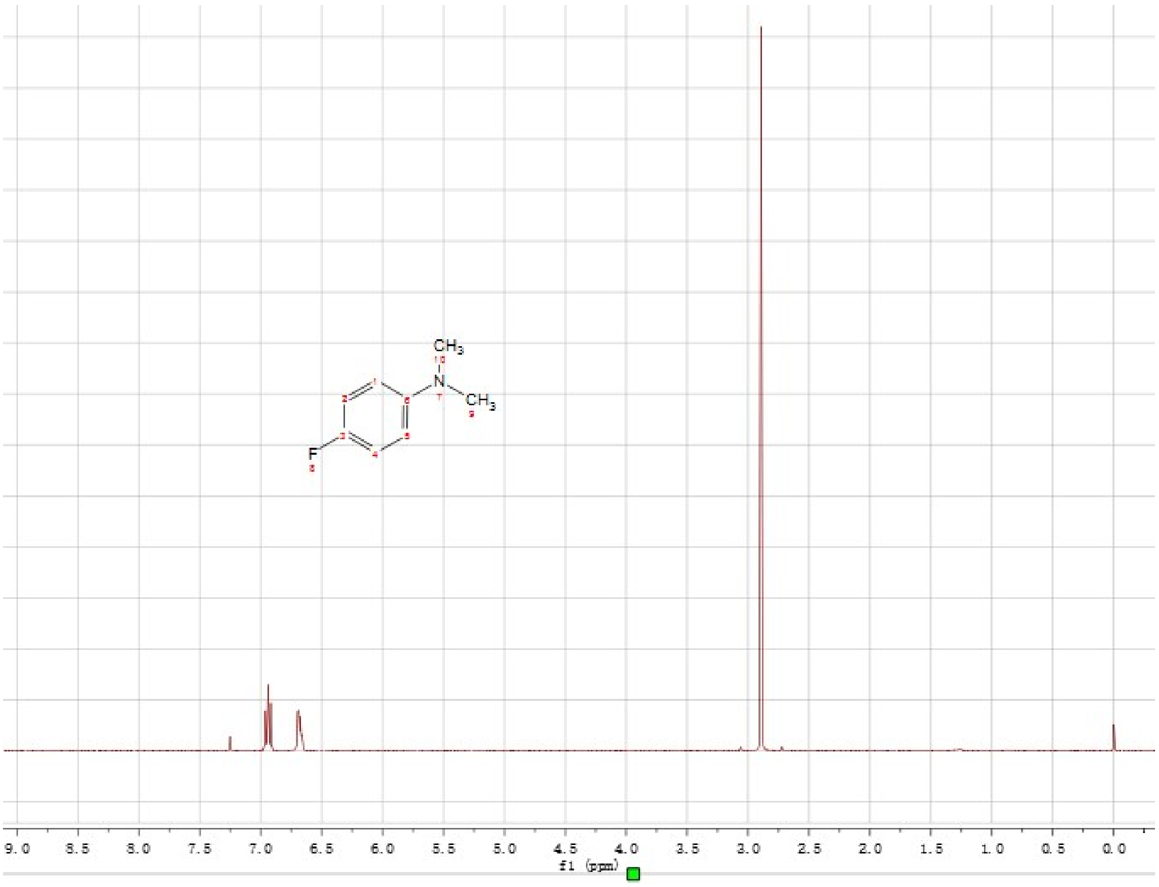

CH3
N
CH3
F
9.0 8.5 8.0 7.5 7.0 6.5 6.0 5.5 5.0 4.5 4.0 3.5 3.0 2.5 2.0 1.5 1.0 0.5 0.0
f1 (ppm)

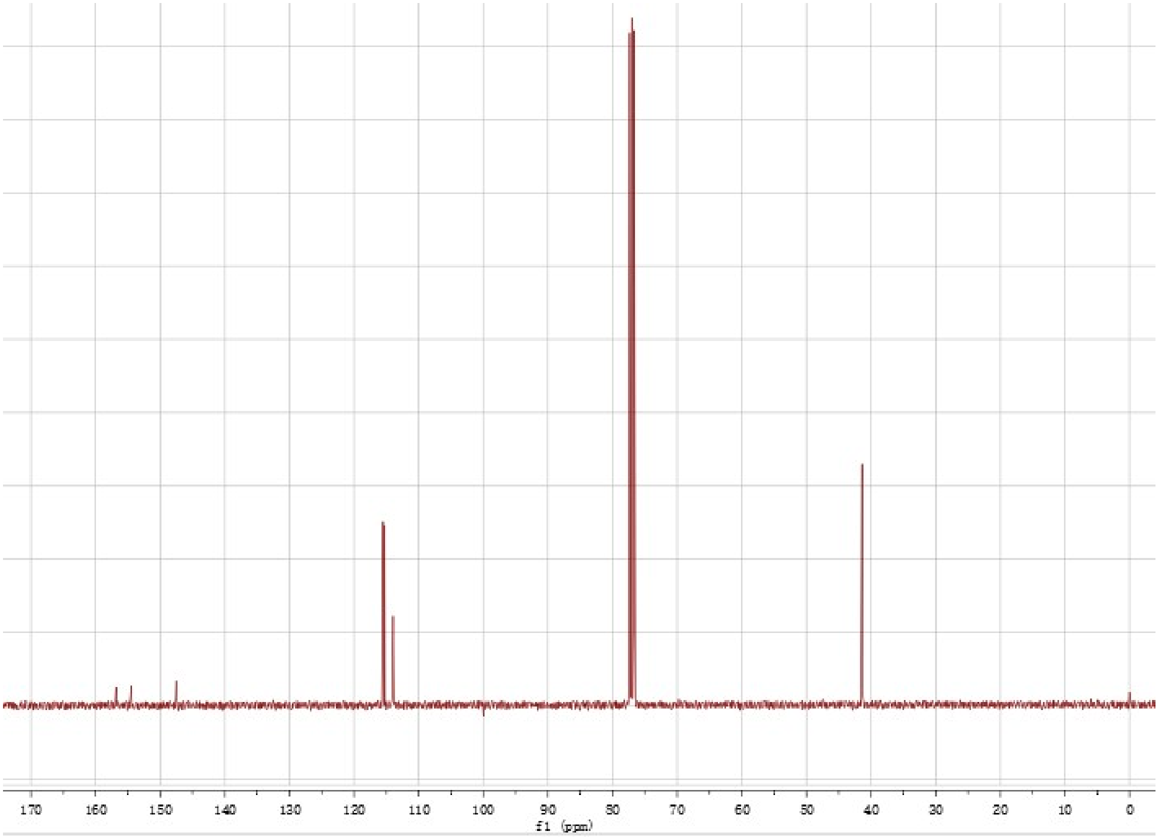

170 160 150 140 130 120 110 100 90 80 70 60 50 40 30 20 10 0
f1 (ppm)

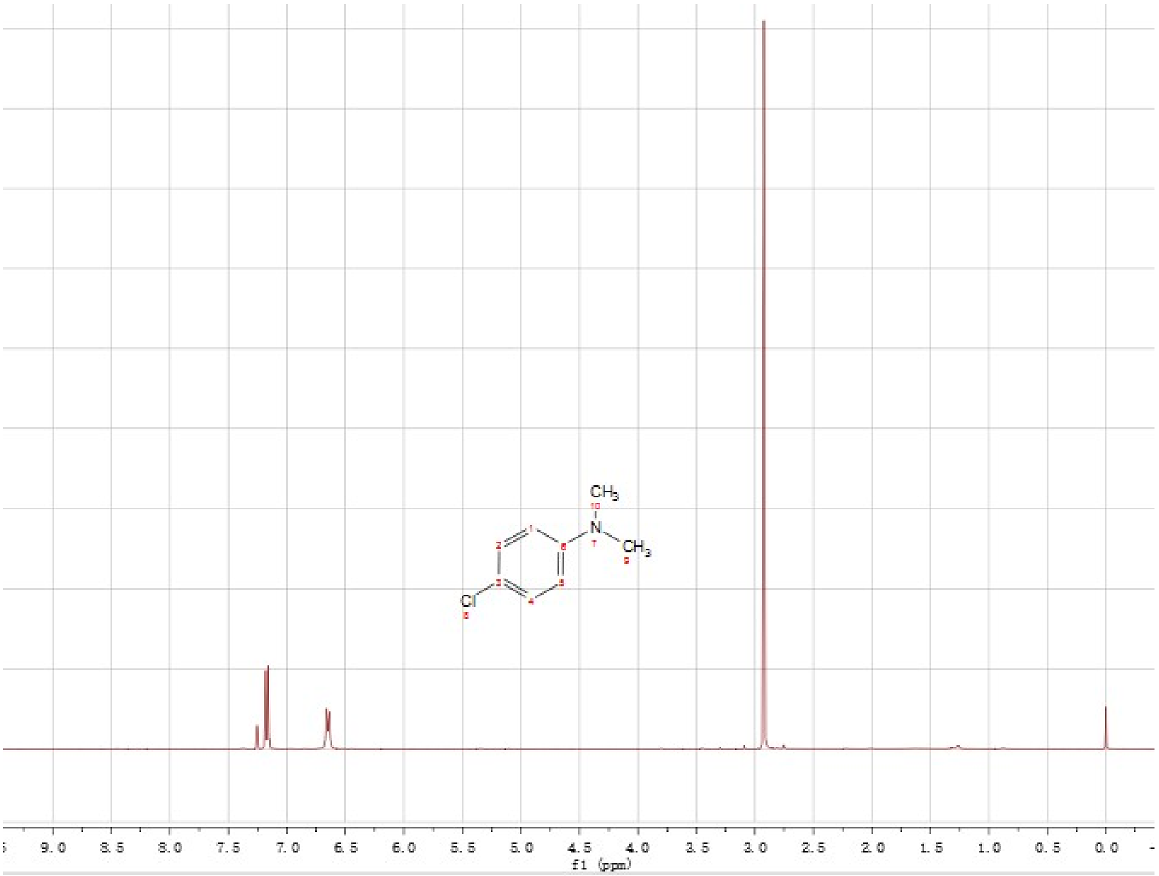
CH3
N
CH3
Cl
9.0 8.5 8.0 7.5 7.0 6.5 6.0 5.5 5.0 4.5 4.0 3.5 3.0 2.5 2.0 1.5 1.0 0.5 0.0
f1 (ppm)

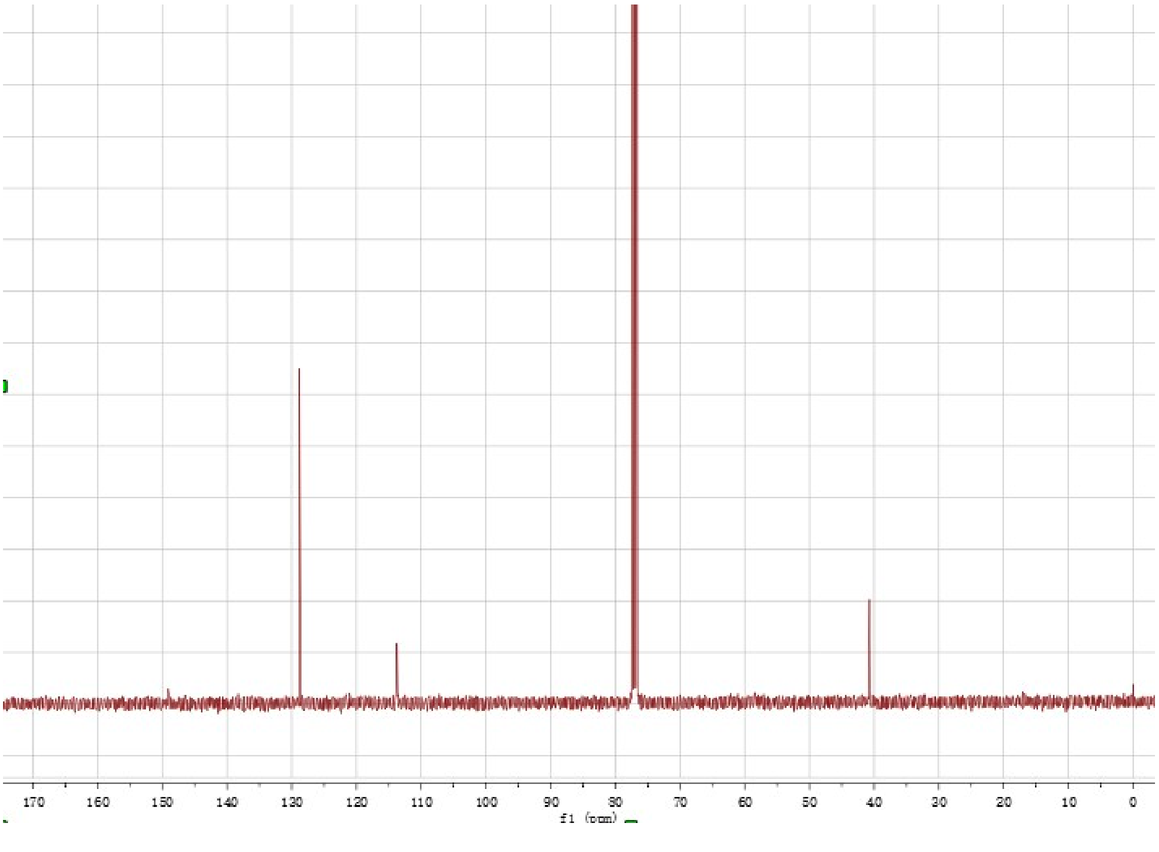
170 160 150 140 130 120 110 100 90 80 70 60 50 40 30 20 10 0
f1 (ppm)

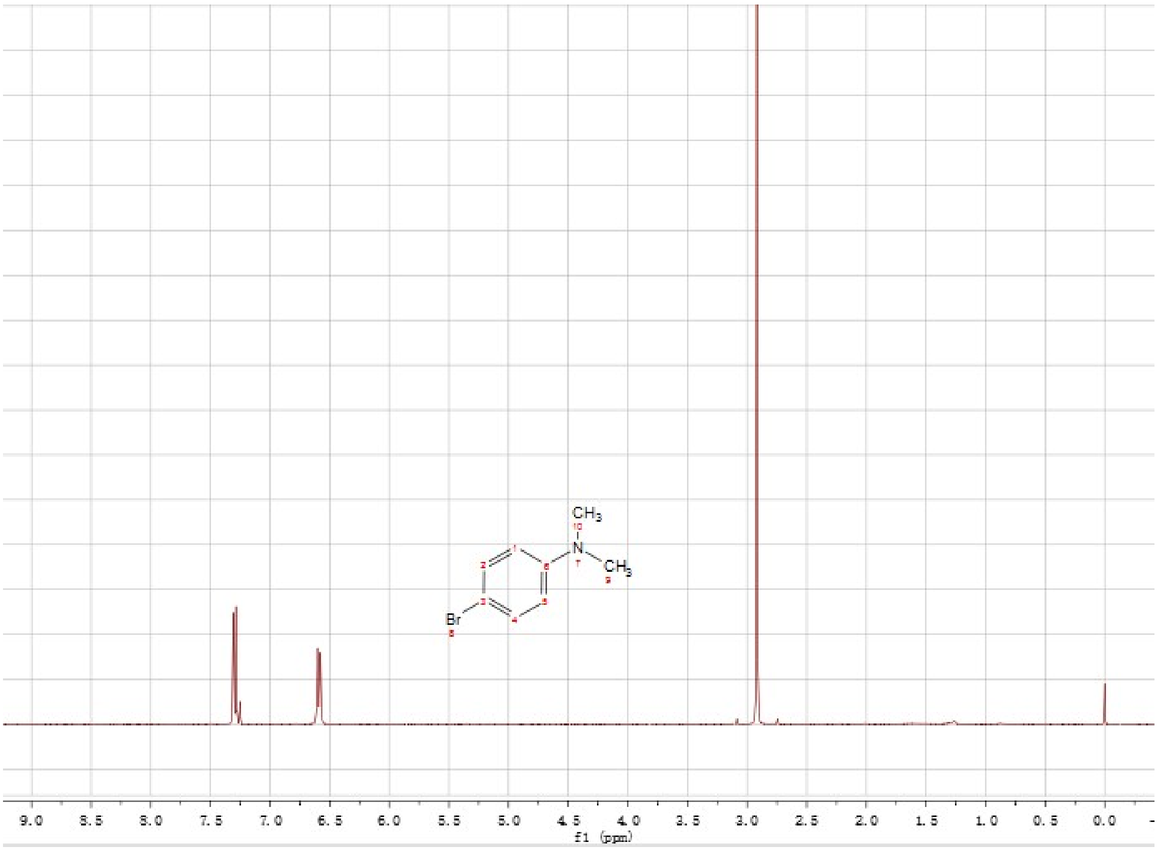
CH3
N
CH3
Br
9.0 8.5 8.0 7.5 7.0 6.5 6.0 5.5 5.0 4.5 4.0 3.5 3.0 2.5 2.0 1.5 1.0 0.5 0.0
f1 (ppm)

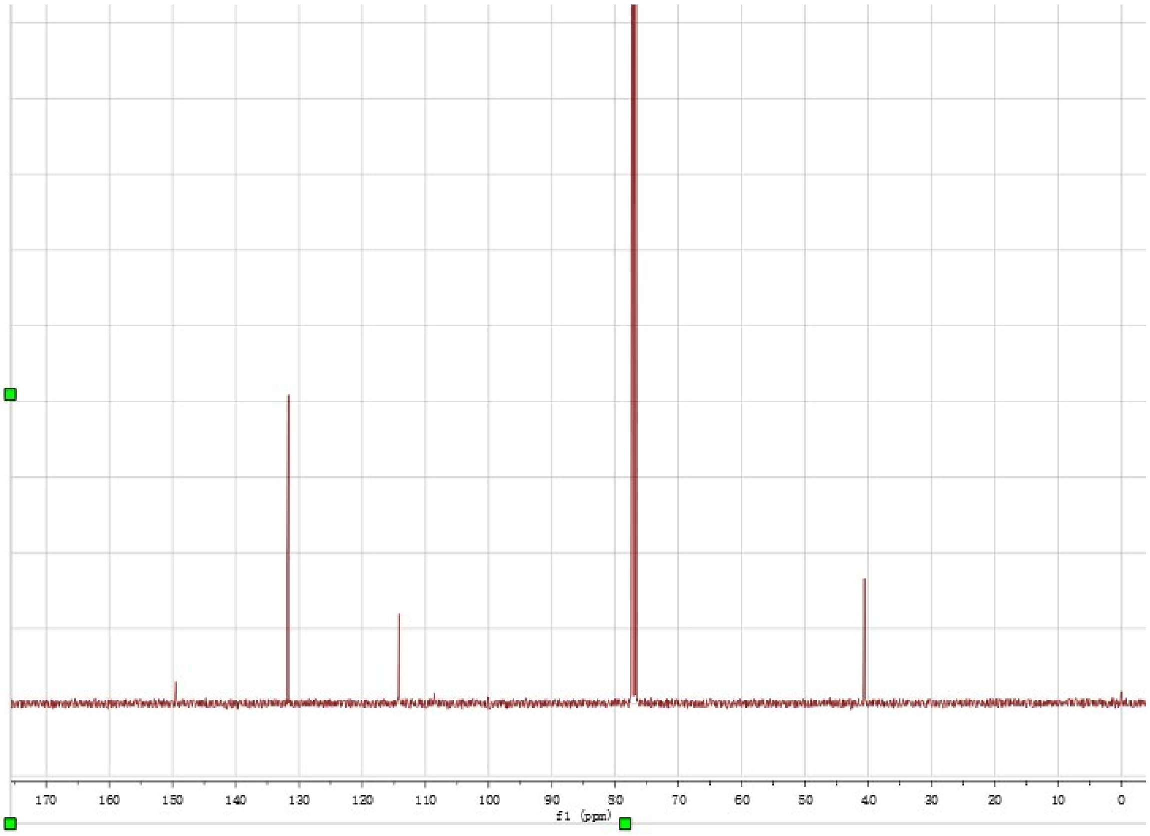
170 160 150 140 130 120 110 100 90 80 70 60 50 40 30 20 10 0
f1 (ppm)

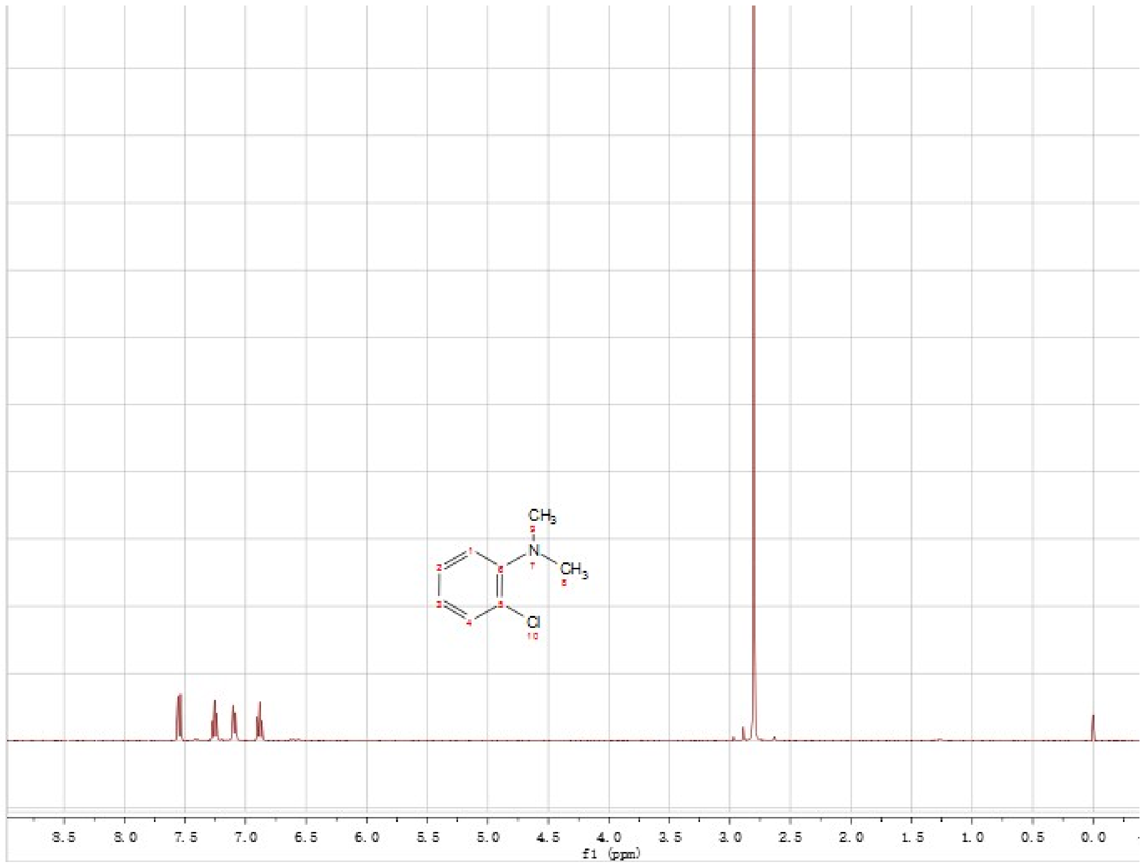
CH3
N
CH3
Cl
8.5
8.0
7.5
7.0
6.5
6.0
5.5
5.0
4.5
4.0
3.5
3.0
2.5
2.0
1.5
1.0
0.5
0.0
f1 (ppm)

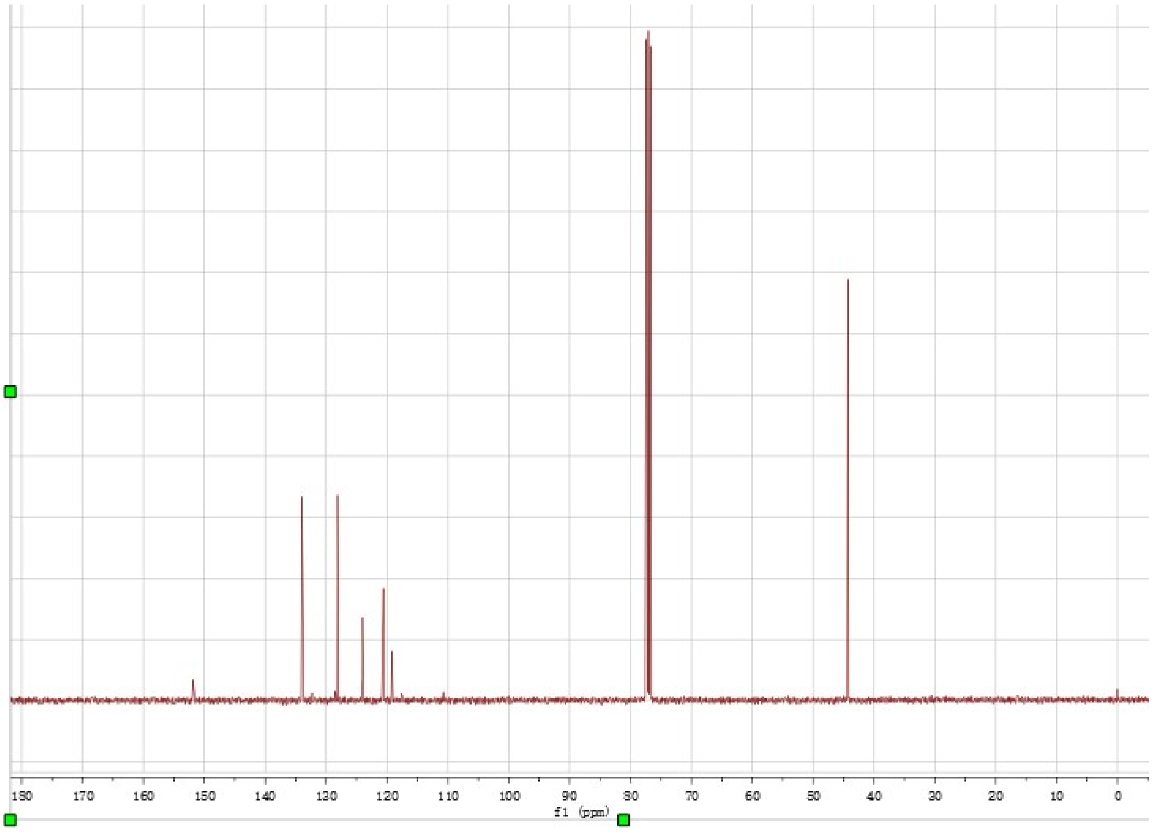
180
170
160
150
140
130
120
110
100
90
80
70
60
50
40
30
20
10
0
f1 (ppm)

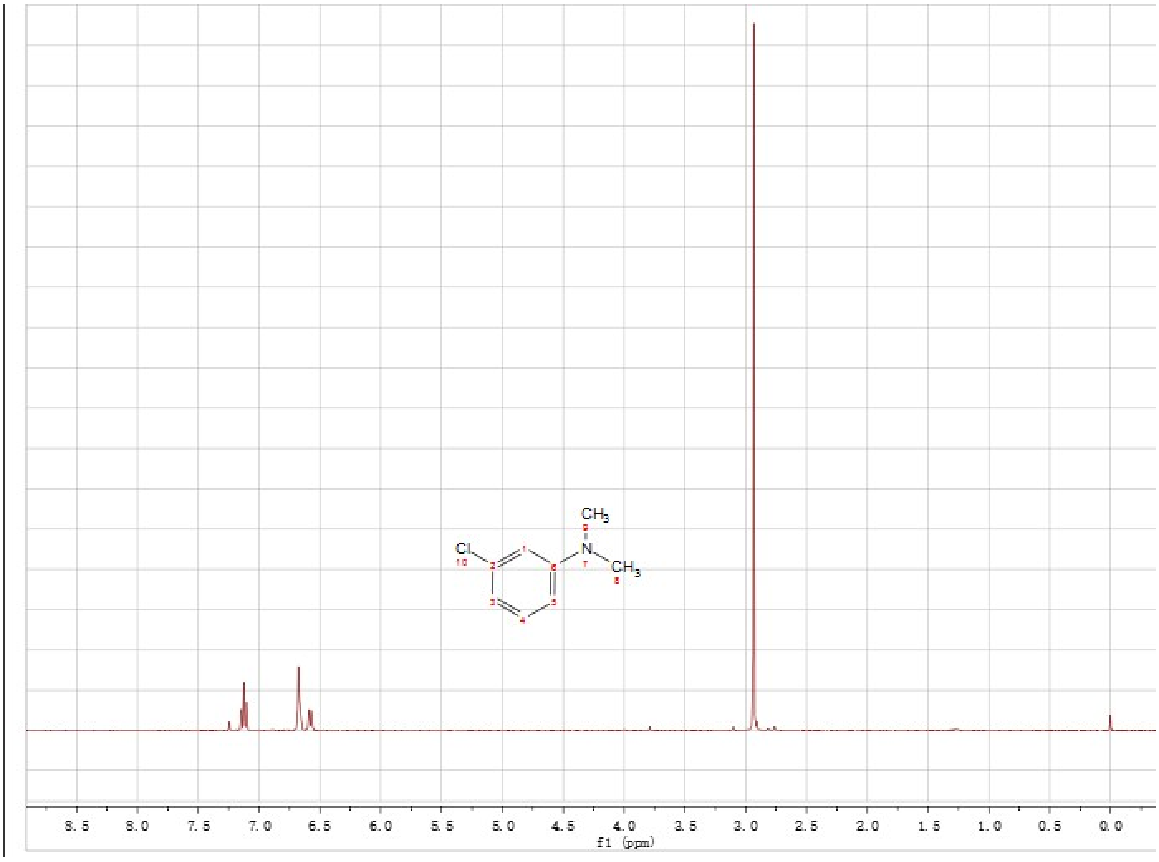
CH3
Cl
N
CH3
8.5
8.0
7.5
7.0
6.5
6.0
5.5
5.0
4.5
4.0
3.5
3.0
2.5
2.0
1.5
1.0
0.5
0.0
f1 (ppm)

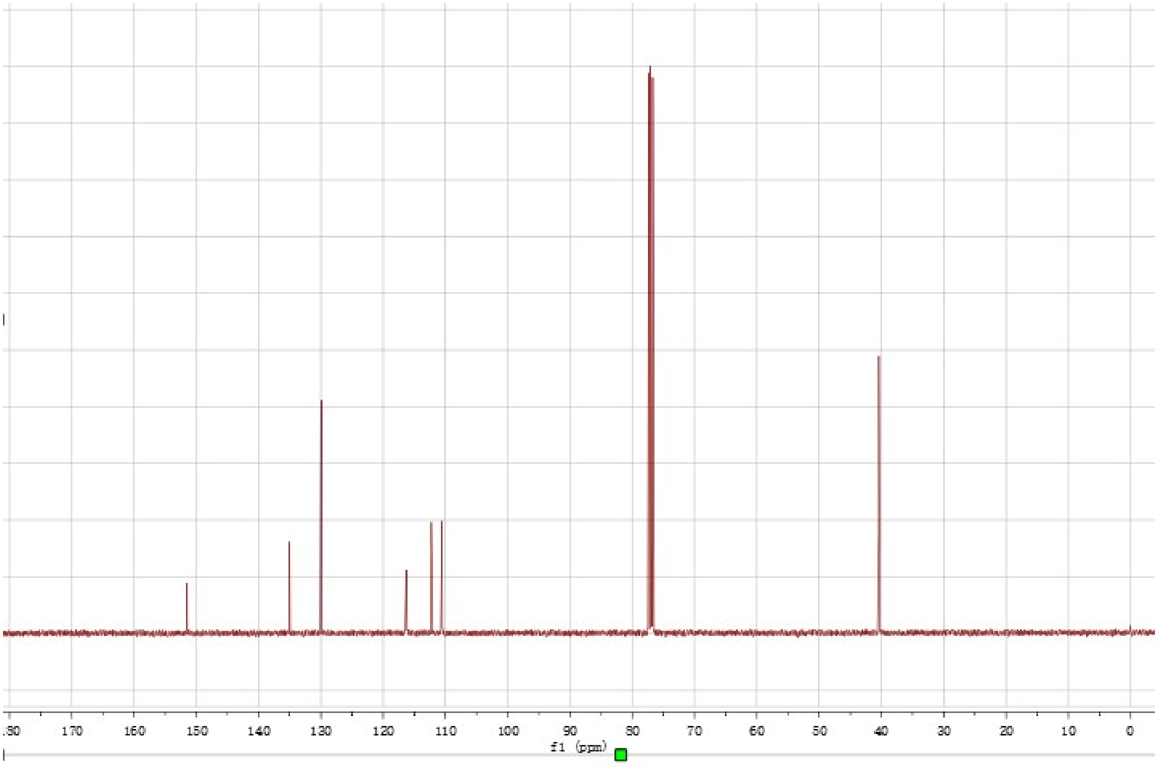
170
160
150
140
130
120
110
100
90
80
70
60
50
40
30
20
10
0
f1 (ppm)

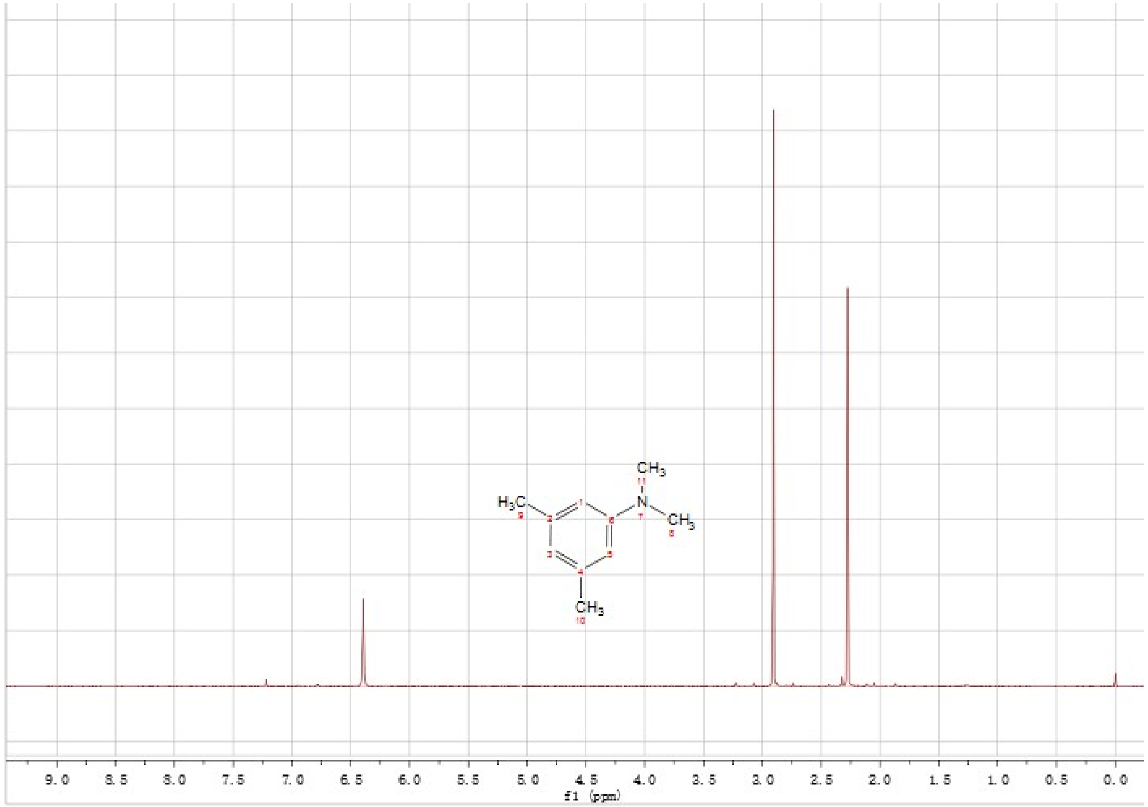

9.0
8.5
8.0
7.5
7.0
6.5
6.0
5.5
5.0
4.5
4.0
3.5
3.0
2.5
2.0
1.5
1.0
0.5
0.0
f1 (ppm)

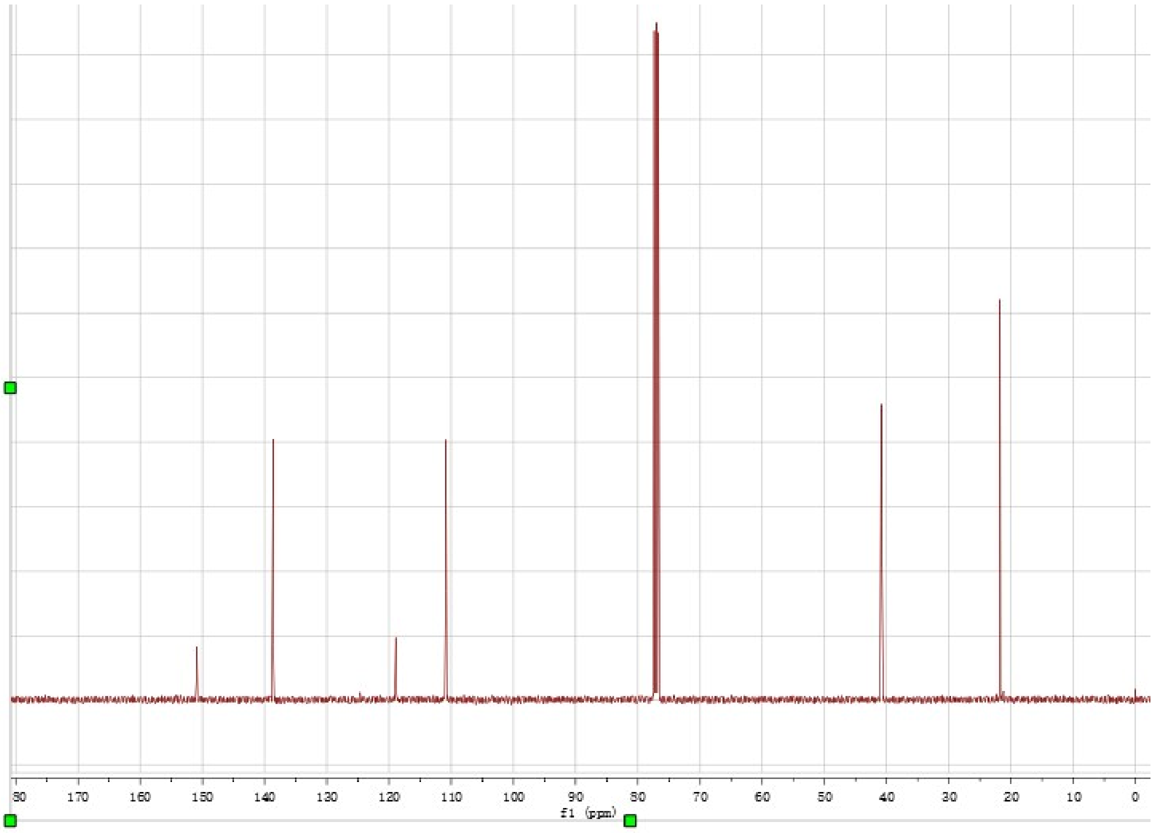

170
160
150
140
130
120
110
100
90
80
70
60
50
40
30
20
10
0
f1 (ppm)
